# Association between parental ADHD and adverse offspring outcomes: Evidence from a nationwide Swedish register-based study

**DOI:** 10.1038/s41380-026-03770-6

**Published:** 2026-07-27

**Authors:** Honghui Yao, Mengping Zhou, Isabell Brikell, Brian M. D’Onofrio, Ralf Kuja-Halkola, Lin Li, Paul Lichtenstein, Henrik Larsson, Erik Pettersson, Zheng Chang

**Affiliations:** 1 Department of Psychiatry, National Clinical Research Center for Mental Disorders, The Second Xiangya Hospital of Central South University, Changsha, 410011 Hunan China; 2 Department of Medical Epidemiology and Biostatistics, Karolinska Institutet, Stockholm, Sweden; 3 Beijing International Center for Mathematical Research, Peking university, Beijing, China; 4 Chongqing Taiji Industry (Group) Co. Ltd, Sinopharm, Chongqin, China; 5 School of Medical Sciences, Örebro University, Örebro, Sweden; 6 Department of Psychological and Brain Sciences, Indiana University, Bloomington, IN USA

**Keywords:** Psychiatric disorders, ADHD

## Abstract

The potential associations between parental ADHD and a broad spectrum of offspring health outcomes remain uncertain. We explored associations between parental ADHD and multiple adverse outcomes in offspring. We used linked Swedish national registers to identify all individuals born in Sweden between 2000 and 2014 (*N* = 1,164,580) and followed them from birth until emigration, death, or December 31, 2020. After matching, 56,853 individuals with parental ADHD and 544,716 individuals without parental ADHD were included in the analysis. Parental ADHD was identified through ADHD diagnoses or ADHD medication prescriptions. Offspring of parents with ADHD were matched to ten individuals whose parents had no ADHD diagnosis. Stratified Cox regression models with robust standard errors were used to estimate associations between parental ADHD and 24 offspring outcomes. Offspring of parents with ADHD had higher rates of most adverse outcomes, compared with those without parental ADHD. Parental ADHD was associated with an increased rate of offspring psychiatric disorders (range of hazard ratios [HRs]: 1.49 for intellectual disability – 4.47 for ADHD), behavioral outcomes (range of HRs: 1.08 for falls – 1.82 for assault/victimization), and somatic disorders (range of HRs: 1.04 for type 1 diabetes–2.47 for sleep disorder). After adjusting for offspring lifetime ADHD, associations weakened but remained significant, except for intellectual disability and epilepsy. Rates were elevated for both maternal and paternal ADHD and highest when both parents with ADHD. Parental ADHD was associated with elevated risks of a broad range of adverse outcomes in offspring, encompassing psychiatric, behavioral, and somatic conditions. These findings highlight the broad intergenerational impact of ADHD and the need for early identification and family-focused preventive strategies.

## Introduction

Attention-deficit/hyperactivity disorder (ADHD) is a common neurodevelopmental disorder characterized by inattention, hyperactivity, and impulsivity [1]. Recent years have seen a rise in adult ADHD diagnoses, partly driven by changing diagnostic criteria and greater awareness of the condition in adulthood [2]. Approximately 3·5% to 5% of adults aged 25 to 34—the typical age range for first-time parenthood—are diagnosed with ADHD [3, 4], and the proportion of children with at least one parent diagnosed with ADHD has notably increased between 2006 and 2016 [5]. These trends underscore the public health importance of better understanding the intergenerational impact of ADHD.

Genetic studies have estimated the heritability of ADHD at approximately 80% [6], and the association between parental and offspring ADHD is likely similarly driven primarily by genetic factors [7]. Beyond direct genetic transmission, recent studies suggest that higher genetic liability for ADHD is modestly associated with certain birth-related characteristics, somatic health conditions, and broader psychosocial adversity linked to ADHD risk [8]. Besides, other factors related to parental ADHD, such as harsh and lax parenting [9], communicating negatively [10], could be the risk factors of ADHD in their offspring. Nevertheless, the association of parental ADHD with other adverse outcomes in offspring is less clear. Observational studies suggest that offspring of parents with ADHD face increased risks for other psychiatric disorders, such as autism spectrum disorder, major depressive disorder, oppositional defiant disorder, and bipolar disorder [11–13]. Additionally, offspring of parents with ADHD may experience behavioral problems, including self-injurious behaviors [14], and somatic health conditions, such as obesity [15] and atopic dermatitis [16].

Despite these findings, existing research has notable limitations, including recall bias, small sample sizes, and limited representativeness [7, 12–15, 17]. Moreover, previous studies have largely focused on psychiatric outcomes, leaving behavioral and somatic health outcomes relatively understudied [7, 11, 13, 14, 17]. Additionally, potential differences between maternal and paternal ADHD in their associations with outcomes in their offspring have not been adequately explored. Biological and caregiving differences between mothers and fathers, such as maternal intrauterine environment, early-life nurturing, and mitochondrial inheritance—might contribute to distinct risk patterns [18]. Furthermore, previous studies have not thoroughly examined the extent to which associations between parental ADHD and outcomes in their offspring are explained by ADHD in the offspring themselves.

This study aimed to comprehensively assess the associations between parental ADHD and a broad range of adverse outcomes in their offspring, including psychiatric, behavioral, and somatic outcomes. We further assessed whether these associations varied according to whether the mother, father, or both parents had ADHD, and explored the potential role of the offspring’s own ADHD in these associations.

## Methods

### Study design and population

We used data from Swedish national registries linked via unique personal identification numbers. The study included data from the following sources: The Medical Birth Register (MBR) includes detailed maternal health and birth outcome data for over 98% of Swedish births since 1973 [19]. The National Patient Register (NPR) contains diagnostic records from inpatient care since 1973 (with complete coverage since 1987), and outpatient visits since 2001, coded using the International Classification of Diseases (ICD) [20]. The Multi-Generation Register includes family relationships among residents since 1932, reliably capturing maternal (97%) and paternal (95%) data for index cases [21]. The Prescribed Drug Register covers all dispensed medications at pharmacies since July 2005, classified by Anatomical Therapeutic Chemical (ATC) codes [22]. The Integrated Database for Labour Market Research (LISA) includes annual socioeconomic data on income, education, and occupation since 1990 for individuals aged 16 years or older [23]. The Cause of Death Register records mortality data with ICD-coded causes since 1961 [24]. The Total Population Register contains demographic and migration information for all residents [25].

**Fig. 1:**
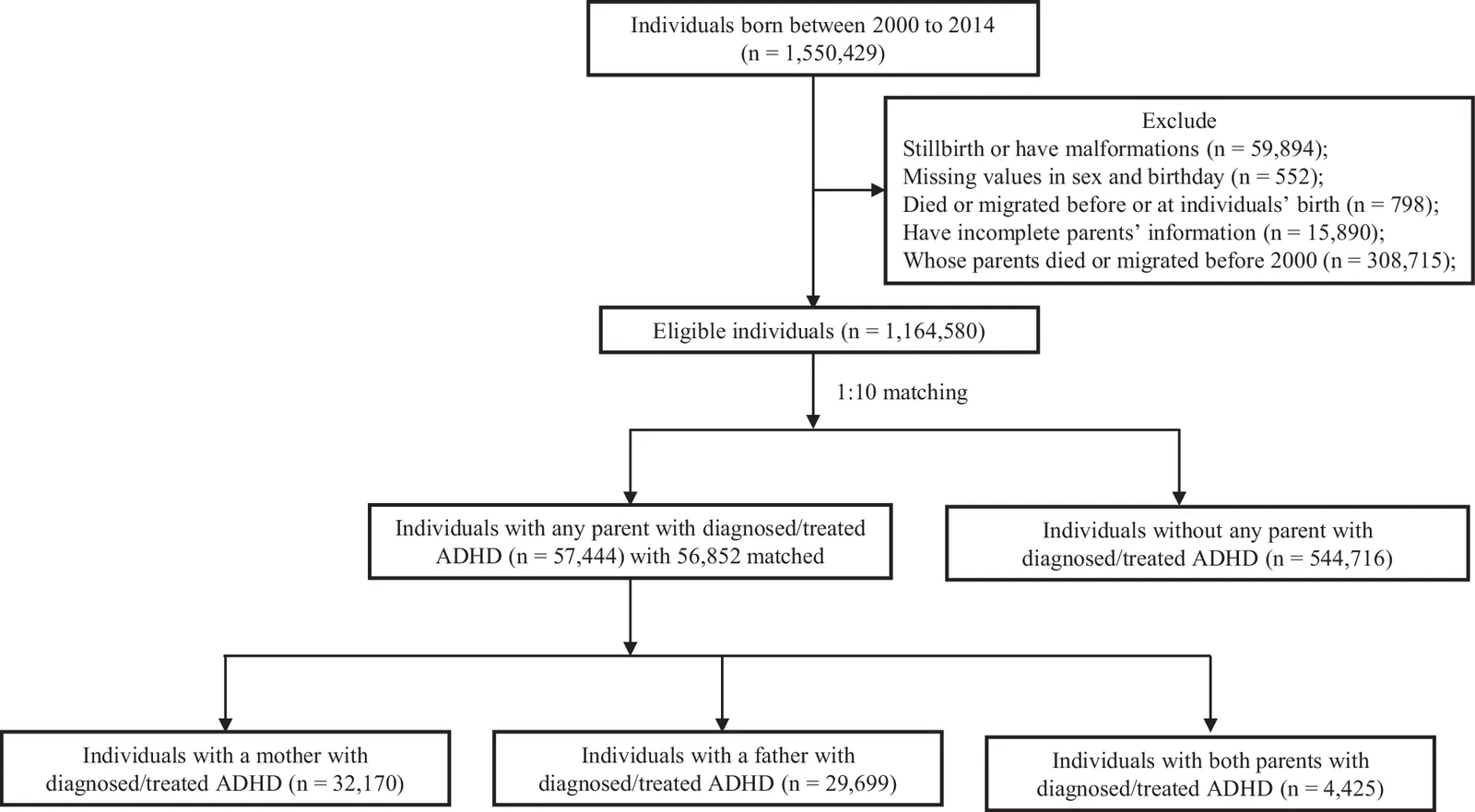
Flowchart of the study cohort.

This cohort comprised all individuals born in Sweden between 2000 and 2014 (*N* = 1,550,429). We linked each individual to their biological parents via the Multi-Generation Register. We excluded stillbirths; individuals with major congenital malformations; individuals with missing sex or date of birth; and individuals for whom either parent had died or emigrated on or before their birth. Additional exclusions included offspring with incomplete parent information or whose parents died or emigrated before 2000. Figure 1 presents the cohort selection flowchart.

### Exposure and outcomes

Parental ADHD was defined as lifetime and identified using data from the NPR and the Prescribed Drug Register, based on ADHD diagnosis (ICD-9: 314; ICD-10: F90) or ADHD medication (ATC codes: N06BA01, N06BA02, N06BA04, N06BA09, N06BA12, C02AC02) [26]. In the main analysis, we considered individuals with any parental ADHD as exposure. In secondary analyses, we further categorized exposure into maternal ADHD only, paternal ADHD only, and both parents diagnosed with ADHD.

We examined 24 adverse outcomes in the offspring in three categories: psychiatric disorders, behavioral outcomes, and somatic diseases. These outcomes were selected based on their clinical relevance to ADHD, evidence from previous literature, and the prevalence of disease in the study age range (6-20 years old). Psychiatric disorders included ADHD, autism, conduct disorders, intellectual disabilities, tic disorders, anxiety, and major depressive disorder. Behavioral outcomes encompassed accidental injuries (including deaths), assaults, falls (including deaths), intentional self-harm, poisoning (including deaths), suicidal behaviors (attempts and suicides), transport injuries (including deaths), and traumatic brain injuries. The somatic diseases category covered asthma, autoimmune diseases, bacterial infections, epilepsy, gastrointestinal autoimmune diseases, obesity, sleep disorders, type 1 diabetes, and viral infections. Detailed ICD codes are presented in Supplementary Table 1.

Because the exposed and unexposed groups differed in key demographic and design characteristics, we used a matched design to examine the associations between parental ADHD and adverse health outcomes in their offspring. Each offspring with any parent with diagnosed/treated ADHD was matched up to ten offspring whose parents had not been diagnosed/treated for ADHD, by offspring sex, birth year, and parental birth years and sex. Controls were randomly selected with replacement within the matching strata. Follow-up began at birth and ended at the individuals’ death, emigration, or on December 31, 2020, whichever occurred first. The final study cohort included 1,164,580 offspring, of whom 32,170 were exposed to maternal ADHD, 29,699 to paternal ADHD, and 4425 to ADHD in both parents.

### Covariates

Covariates included parental history of psychiatric comorbidities (substance use disorder, schizophrenia, bipolar disorder, major depressive disorder, obsessive-compulsive disorder, personality disorders, post-traumatic stress disorders, and anxiety disorders), identified from the NPR using ICD codes (Supplementary Table 1) and coded as a binary variable indicating the presence of any psychiatric comorbidity before the offspring’s birth. Parental education levels (categorized as primary/lower secondary, upper secondary, post-secondary/postgraduate, or unknown) were obtained from the LISA database at the birth of their offspring.

### Statistical analysis

We evaluated the associations between parental ADHD and adverse outcomes in their offspring using stratified Cox regression models, with strata defined by matched sets, and reported adjusted hazard ratios (HRs) with 95% confidence intervals (CIs). The underlying time scale was time since birth of the offspring, and incidence rates were reported per 1000 person-years. Robust standard errors accounted for within-family correlations. Analyses were adjusted for parental psychiatric comorbidities and education levels. We further adjusted for lifetime ADHD diagnosis in the offspring to assess whether the associations between parental ADHD and adverse outcomes were explained by the offspring’s ADHD. We applied a Bonferroni correction to account for multiple testing across the 96 analyses, corresponding to 24 outcomes examined across four exposure groups (any parent with ADHD, maternal ADHD only, paternal ADHD only, and both parents with ADHD). An adjusted P-value of 0.0005 was considered statistically significant. Subgroup analyses were conducted by offspring sex (male vs. female) and by parental birth year (before 1980 vs. 1980 and later), separately for mothers and fathers.

Sensitivity analyses were conducted to assess the robustness of the findings. First, because exposure misclassification could arise from differences in how parental ADHD is defined, we conducted a sensitivity analysis in which parental ADHD was defined solely based on diagnoses recorded in the NPR to assess whether the choice of definition influenced the results. Second, because ADHD was not formally recognized in diagnostic manuals until the late 1980s, and the Swedish registers have more complete coverage of ADHD diagnoses for later birth cohorts, we restricted analyses to individuals whose parents were born after 1985. This approach improves ascertainment of parental ADHD and reduces the risk of exposure misclassification. Third, to explore the potential influence of parental ADHD timing in relation to offspring development, we redefined exposure according to whether the parent had any ADHD diagnosis or medication record after the offspring’s birth, which may better reflect potential ongoing parental ADHD during the child’s upbringing, or only before childbirth.

Data management was executed using SAS (Version 9.4, SAS Institute Inc.), and statistical analyses were performed in R (Version 4.3.2). The reporting of this study followed the Strengthening the Reporting of Observational Studies in Epidemiology (STROBE) guidelines [27].

## Results

We identified 1,164,580 eligible individuals born between 2000 and 2014 (Fig. 1). Among these, 57,444 (4.9%) individuals had at least one parent diagnosed with ADHD. After matching, our analysis included 56,852 individuals exposed to parental ADHD (52.5% male). The majority were born between 2007 and 2014 (61.1%) (Table 1).

**Table 1:** Baseline characteristics of the study population after matching.

	Any parent with ADHD	No parent with ADHD
**Children characteristics**		
Sex		
Males	29,836 (52.5%)	286,168 (52.5%)
Females	27,016 (47.5%)	258,548 (47.5%)
Birthday year		
2000 - 2006	22,214 (39.1%)	211,907 (38.9%)
2007 - 2014	34,638 (60.9%)	332,809 (61.1%)
**Mother characteristics**		
Birthday year		
1950 – 1960	101 (0.2%)	610 (0.1%)
1961 - 1970	5512 (9.7%)	52,076 (9.6%)
1971 - 1980	24,515 (43.1%)	239,245 (43.9%)
After 1980	26,724 (47.0%)	252,785 (46.4%)
Education levels		
Primary and lower secondary education	15,587 (27.4%)	75,391 (13.8%)
Upper secondary education	27,483 (48.3%)	248,433 (45.6%)
Post-secondary and postgraduate education	12,988 (22.9%)	206,954 (38.0%)
Unknown	794 (1.4%)	13,938 (2.6%)
Parental psychiatric comorbidity before the index child’s birth	15,251 (26.8%)	37,825 (6.9%)
**Father characteristics**		
Birthday year		
Before 1950	23 (0.0%)	34 (0.0%)
1950 – 1960	991 (1.7%)	7,235 (1.3%)
1961 - 1970	9415 (16.6%)	89,965 (16.5%)
1971 - 1980	26,739 (47.0%)	262,233 (48.1%)
After 1980	19,684 (34.6%)	185,249 (34.0%)
Education levels		
Primary and lower secondary education	15,377 (27.1%)	78,123 (14.3%)
Upper secondary education	30,862 (54.3%)	291,833 (53.6%)
Post-secondary and postgraduate education	9893 (17.4%)	164,284 (30.2%)
Unknown	720 (1.3%)	10,476 (1.9%)
Parental psychiatric comorbidity before the index child’s birth	11,874 (20.9%)	23,226 (4.3%)

The distribution of parental birth years was similar between exposure groups, with only small differences (e.g., mothers born after 1980: 47·0% in the exposed group vs. 46·4% in the unexposed; fathers born between 1971 and 1980: 47·0% vs. 48·1%). Parents with ADHD had lower educational attainment and a higher prevalence of psychiatric comorbidities (mothers 26·8% vs. 6·9%; fathers 20·9% vs. 4·3%) than parents without ADHD (post-secondary education: mothers 22·9% vs. 38·0%; fathers 17·4% vs. 30·2%). These demographic and socioeconomic patterns were consistent across exposure subgroups (maternal ADHD only, paternal ADHD only, and both parents diagnosed). Additional demographic details by exposure group are provided in Supplementary Table 2.

### Parental ADHD and the absolute rate of adverse outcomes in their offspring

**Fig. 2:**
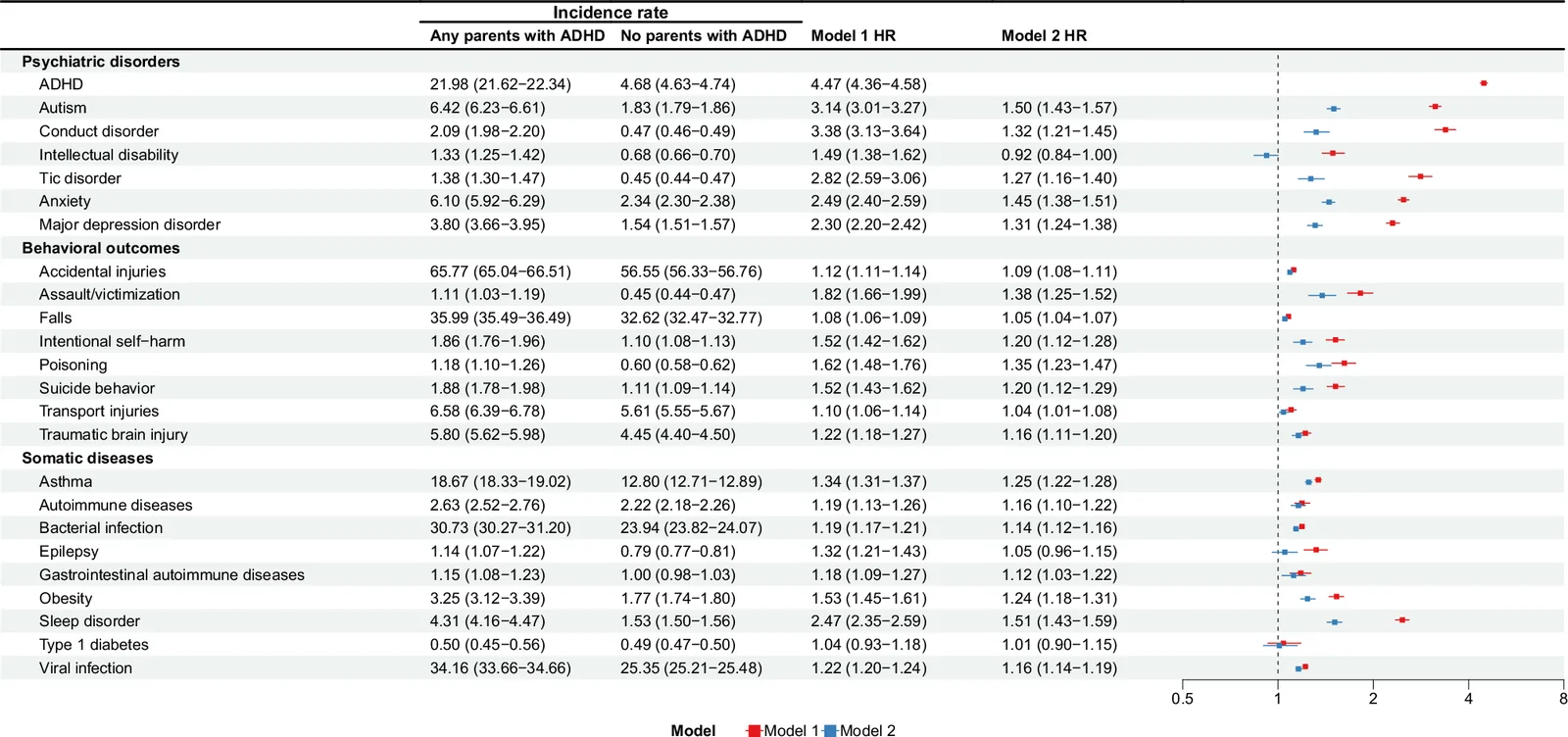
Associations between parental ADHD and offspring adverse outcomes. ADHD Attention-Deficit/Hyperactivity Disorder. Notation: Accidental injuries, falls, poisoning, suicide behavior, and transport injuries include both diagnoses and deaths; all other outcomes are based on diagnoses only. Model 1 adjusted for maternal and paternal education levels, as well as maternal and paternal psychiatric comorbidities. Model 2 additionally adjusted for lifetime ADHD in their offspring. The reference group consists of offspring whose parents both did not have ADHD. Incidence rates were reported per 1000 person-years.

Offsprings of parents with ADHD demonstrated higher incidence rates for all assessed adverse outcomes compared to those without parental ADHD. Among individuals with at least one parent with ADHD, incidence rates ranged from 0·50 per 1000 person-years (type 1 diabetes) to 65·77 per 1000 person-years (accidental injuries) (Fig. 2). ADHD was the most frequently diagnosed psychiatric disorder among individuals with parental ADHD (21·98 per 1000 person-years) compared to non-exposed individuals (4·68 per 1000 person-years). Anxiety was also common, with a higher incidence in individuals with parental ADHD (6·10 vs. 2·34 per 1000 person-years). Accidental injuries and falls were the most common behavioral outcomes, with higher rates in individuals with parental ADHD (65·77 vs. 56·55 and 35·99 vs. 32·62 per 1000 person-years, respectively). Among somatic disorders, type 1 diabetes had the lowest incidence and showed minimal differences between exposed (0·50 per 1000 person-years) and non-exposed groups (0·49 per 1000 person-years), whereas viral infections showed the highest incidence in both the exposed (34·16 per 1000 person-years) and non-exposed groups (25·35 per 1000 person-years). Incidence rates by maternal or paternal ADHD are provided in the Supplementary Table 3. The highest incidence rates for adverse outcomes occurred among individuals with both parents diagnosed with ADHD, followed by maternal-only ADHD.

### Association between parental ADHD and adverse outcomes in their offspring

After adjusting for parental education and psychiatric comorbidities, parental ADHD was consistently associated with elevated rates for most adverse outcomes, except for type 1 diabetes (Fig. 2). The associations remained significant after Bonferroni correction for multiple testing. The associations were observed for psychiatric disorder: HRs ranged from 1·49 (95% CI, 1·38–1·62) for intellectual disability to 4·47 (95% CI, 4·36–4·58) for ADHD. For behavioral outcomes, the associations ranged from 1·08 (95% CI, 1·06–1·09) for falls to 1·82 (95% CI, 1·66–1·99) for assault or victimization. Among somatic conditions, sleep disorders had the strongest significant association (HR, 2·47; 95% CI, 2·35–2·59), while gastrointestinal autoimmune diseases had a weaker but still significant association (HR, 1·18; 95% CI, 1·09–1·27).

Supplementary Table 4 presents the number and proportion of offspring with ADHD diagnosed prior to each outcome among individuals with that outcome. Offspring ADHD was frequently diagnosed before several psychiatric and behavioral outcomes, suggesting that it may partly contribute to the elevated risks observed in these domains. After additional adjustment for offspring ADHD, the associations between parental ADHD and the outcomes were generally attenuated (Fig. 2) but remained significant, except for intellectual disability (HR, 0·92; 95% CI, 0·84–1·00) and epilepsy (HR, 1·04; 95% CI, 0·95–1·13).

Subgroup analyses based on type of parental ADHD exposure (maternal-only, paternal-only, or both parents) showed consistent patterns after adjusting for parental education and psychiatric comorbidities (Table 2) and after further adjusting for ADHD in the offspring (Supplementary Table 5). The highest rates for psychiatric disorders were observed in individuals with both parents diagnosed with ADHD, followed by maternal-only ADHD. Behavioral and somatic outcomes showed minimal variation across these subgroups.

**Table 2:** Associations between parental ADHD and offspring adverse outcomes by parental ADHD status.

	Maternal ADHD	Paternal ADHD	Both parents with ADHD
	Adjusted hazard ratio (95% CI)*	Adjusted hazard ratio (95% CI)*	Adjusted hazard ratio (95% CI)*
**Psychiatric disorders**			
ADHD	4.96 (4.81–5.12)	4.27 (4.13–4.42)	7.05 (6.47–7.67)
Autism	3.58 (3.41–3.77)	2.94 (2.78–3.12)	5.15 (4.51–5.89)
Conduct disorder	3.60 (3.29–3.95)	3.37 (3.04–3.73)	4.98 (3.98–6.24)
Intellectual disability	1.39 (1.25–1.55)	1.64 (1.47–1.82)	1.57 (1.20–2.05)
Tic disorder	3.03 (2.73–3.37)	2.68 (2.38–3.02)	3.66 (2.69–4.97)
Anxiety	2.72 (2.60–2.86)	2.33 (2.21–2.46)	3.35 (2.89–3.87)
Major depressive disorder	2.58 (2.43–2.74)	2.08 (1.94–2.23)	2.76 (2.28–3.34)
**Behavioral outcomes**			
Accidental injuries	1.15 (1.13–1.17)	1.11 (1.09–1.13)	1.17 (1.11–1.23)
Assault/victimization	1.92 (1.71–2.14)	1.83 (1.62–2.07)	2.59 (1.96–3.42)
Falls	1.08 (1.06–1.10)	1.08 (1.05–1.10)	1.09 (1.02–1.16)
Intentional self-harm	1.49 (1.37–1.63)	1.58 (1.45–1.73)	1.94 (1.53–2.46)
Poisoning	1.77 (1.60–1.97)	1.60 (1.42–1.80)	2.69 (2.11–3.42)
Suicide behavior	1.49 (1.37–1.62)	1.60 (1.46–1.74)	1.95 (1.54–2.47)
Transport injuries	1.06 (1.01–1.11)	1.14 (1.09–1.20)	1.03 (0.90–1.17)
Traumatic brain injury	1.26 (1.20–1.32)	1.19 (1.13–1.25)	1.22 (1.08–1.39)
**Somatic diseases**			
Asthma	1.45 (1.41–1.49)	1.24 (1.20–1.28)	1.49 (1.37–1.62)
Any autoimmune diseases	1.26 (1.17–1.35)	1.12 (1.04–1.21)	1.22 (0.99–1.51)
Bacterial infection	1.27 (1.24–1.30)	1.11 (1.08–1.14)	1.19 (1.11–1.28)
Epilepsy	1.26 (1.14–1.41)	1.41 (1.26–1.58)	1.49 (1.11–2.02)
Gastrointestinal autoimmune diseases	1.27 (1.15–1.40)	1.10 (0.98–1.24)	1.31 (0.96–1.80)
Obesity	1.65 (1.55–1.76)	1.37 (1.28–1.48)	1.34 (1.11–1.63)
Sleep disorder	2.71 (2.56–2.87)	2.32 (2.17–2.48)	3.38 (2.87–3.98)
Type 1 diabetes	1.13 (0.97–1.32)	1.00 (0.85–1.19)	1.45 (0.94–2.22)
Viral infection	1.30 (1.27–1.33)	1.14 (1.11–1.17)	1.26 (1.18–1.35)

Notation: Accidental injuries, falls, poisoning, suicide behavior, and transport injuries include both diagnoses and deaths; all other outcomes are based on diagnoses only.

*Models are adjusted for maternal and paternal education levels, as well as maternal and paternal psychiatric comorbidities. The reference group consists of offspring whose parents both do not have ADHD.

*ADHD* Attention-Deficit/Hyperactivity Disorder.

Sex-stratified analyses indicated that the associations between parental ADHD and adverse outcomes in their offspring were generally stronger among females compared to males in most psychiatric disorders (Supplementary Figures 1–4). Individuals whose any parent with ADHD were significantly associated with an increased risk of transport injuries only among females (HR, 1·18; 95% CI, 1·12–1·23), whereas the association with gastrointestinal autoimmune diseases was significant only among males (HR, 1·34; 95% CI, 1·19–1·51). In analyses stratified by parental birth year, parents born before 1980 exhibited stronger associations with several psychiatric outcomes, assault/victimization, and gastrointestinal autoimmune diseases (Supplementary Figures 5, 6).

Sensitivity analyses indicated robustness of the primary findings. Redefining parental ADHD by diagnoses only did not substantially alter the results (Supplementary Table 6). Similarly, analyses limited to individuals whose parents were born after 1985 yielded results consistent with the primary analyses, except for no significant associations for transport injuries and gastrointestinal autoimmune diseases (Supplementary Table 7). Analyses in which parental ADHD was defined based on diagnoses or medication use occurring after the offspring’s birth yielded results broadly consistent with the main findings (Supplementary Table 8). In contrast, when parental ADHD was restricted to diagnoses or medication records occurring only before the offspring’s birth, the increased risks for neurodevelopmental disorders remained, whereas many associations for behavioral and somatic outcomes were no longer statistically significant (Supplementary Table 9), which may be partly attributable to the smaller sample size or to a direct parental effect.

## Discussion

Using nationwide health registers, we found that parental ADHD was associated with a broad range of adverse psychiatric, behavioral, and somatic outcomes in their offspring. The associations were observed for both maternal and paternal ADHD and were strongest when both parents had ADHD. After adjustment for offspring ADHD diagnosis, these associations were generally attenuated but remained significant for most outcomes. These findings highlight the broad intergenerational impact of ADHD and underscore the importance of early identification and family-focused preventive strategies to mitigate adverse outcomes in offspring.

Although this study was not designed to examine underlying mechanisms, several pathways may be relevant for the associations across outcome domains. First, shared familial liability, including genetic predisposition to ADHD and related disorders, is likely to contribute to the clustering of adverse outcomes in offspring [13, 28, 29]. Second, broader environmental and social factors linked to parental ADHD, such as socioeconomic disadvantage [30] and stigma [31], may further contribute to their offspring vulnerability. Third, Parental ADHD may also influence the family environment through challenges in parenting, including difficulties maintaining consistent supervision, safety practices, and daily routines, which may increase the risk of adverse outcomes in their offspring [32–35]. These pathways likely act in combination across outcome domains.

Consistent with previous research, our findings indicate that parental ADHD is associated with increased risks of multiple psychiatric disorders in offspring, including ADHD [12], autism [36, 37], conduct disorders [13], anxiety [13], and major depressive disorder [11]. Extending previous work, we also observed an increased risk of tic disorders in offspring and further compared associations across maternal, paternal, and dual-parent ADHD. Notably, adjustment for offspring ADHD attenuated these associations, suggesting that shared familial liability or pathways involving offspring ADHD partly account for the observed risks. We further showed that diagnosis of ADHD in the offspring frequently preceded several psychiatric and behavioral outcomes. However, because ADHD onset often precedes clinical diagnosis, these temporal patterns should be interpreted as ordering of recorded diagnoses rather than definitive evidence about the sequence of underlying symptom development.

Parental ADHD was also associated with increased risks of adverse behavioral outcomes in offspring, with particularly strong associations observed for self-harm, suicide, and assault/victimization [32]. These findings are clinically and publicly important, as they point to substantial psychosocial vulnerability among offspring of parents with ADHD. Compared with several unintentional injury outcomes, these stronger associations may reflect the contribution of persistent emotional, behavioral, and social difficulties [38]. These associations were attenuated after adjustment for offspring ADHD, suggesting that offspring ADHD may partly contribute to the elevated risks in these domains but is unlikely to fully account for them.

In somatic health domain, parental ADHD was associated with elevated risks of several conditions, including asthma, any autoimmune diseases, bacterial infection, gastrointestinal autoimmune diseases, obesity, sleep disorder, viral infection. The observed association with obesity is consistent with previous research linking parental ADHD to metabolic risk in children [15], while associations with other somatic disorders are novel. No significant association between parental ADHD and type 1 diabetes in their offspring, which aligns with existing evidence showing no link between parental neurodevelopmental disorders and type 1 diabetes [39]. We also observed relatively strong associations between parental ADHD and offspring sleep disorders. Because our outcome captured a broad category of clinically recorded sleep disorders, the strong association observed may indicate that sleep problems are an important component of the broader familial vulnerability associated with parental ADHD, rather than an isolated comorbidity [40].

We found that the associations between parental ADHD and offspring outcomes were generally similar for maternal and paternal ADHD, while the highest risks were observed when both parents had ADHD. The similarity between maternal and paternal associations may suggest that shared familial mechanisms, including genetic liability and broader family environmental factors, play an important role across outcome domains. The higher risks observed when both parents had ADHD may reflect the combined influence of greater genetic liability, and increased environmental challenges such as greater co-parenting stress, unequal caregiving responsibilities, and heightened family conflict [41]. Families in which both parents are affected may therefore benefit more from closer monitoring and targeted support. We also observed limited sex differences in the cross-generational effects of parental ADHD, which may suggest that intergenerational mechanisms, such as shared genetic liability and family environmental factors, operate similarly in males and females.

To our knowledge, this is the first large-scale population-based study to comprehensively examine the associations between parental ADHD and a broad spectrum of offspring outcomes. Nevertheless, these findings should be interpreted in the context of several limitations. First, parental and offspring diagnoses were based on specialist care and prescribed medication records; thus, individuals managed solely in primary care or those who did not seek treatment were not captured. This may have led to underestimation of incidence rates, especially for behavioral and somatic outcomes. Second, because parental ADHD was identified through diagnosis records, misclassification is possible, especially in older parental cohorts with lower likelihood of diagnosis. However, sensitivity analyses restricted to offspring whose parents were born after 1985 generated similar results. Third, the study used diagnostic codes from three different ICD versions (ICD-8, ICD-9, and ICD-10), and changes in diagnostic criteria and clinical practices over time could have introduced inconsistencies in disease classification. We managed to reduce this potential bias by matching exposed and unexposed individuals on both parental and offspring birth year. Fourth, because parental birth year was included in the matching procedure, we were not able to examine parental age as an independent factor. Although parental age has been widely studied in relation to offspring outcomes [11], its relevance in the context of parental ADHD may merit further investigation. Fifth, the registers lacked detailed information on paternal co-residence or caregiving involvement, particularly for non-cohabiting parents, limiting our ability to disentangle parenting-related environmental pathways from other familial influences. Finally, although adjustment for offspring ADHD and the timing-based sensitivity analyses provided additional insight, our design does not allow causal disentangling of genetic liability, offspring-related pathways, and environmental influences.

Although our study highlights the broad intergenerational impact of parental ADHD, a few subsequent questions remain regarding the underlying mechanisms and potential interventions. Because of high heritability of ADHD, future research could also move beyond documenting associations to examine the interplay between genetic liability and modifiable environmental influences, including parenting, school context, and peer relationships. The timing-based sensitivity analyses suggest that the presence of parental ADHD across the child’s development may be relevant, particularly for behavioral and somatic outcomes. Addressing these questions will require genetically informative longitudinal cohort studies with richer measures of family and social environments than are typically available in register-based studies. Such studies could also examine the course of parental ADHD over time, including persistence, remission, and treatment, to clarify whether the timing and continuity of parental ADHD are differentially related to offspring outcomes. Resilience-informed longitudinal approaches may also help explain why some offspring of parents with ADHD show adaptive outcomes despite elevated familial risk and may aid in identifying protective factors that could inform preventive interventions [42].

## Conclusion

Parental ADHD was associated with a wide spectrum of adverse outcomes in offspring, spanning psychiatric, behavioral, and somatic domains. These findings underscore the broad intergenerational impact of ADHD, extending beyond the direct transmission of the disorder to a wider constellation of health and developmental vulnerabilities in the next generation. As ADHD diagnoses become increasingly common among adults of reproductive age, recognizing and addressing the needs of families affected by parental ADHD will be essential for promoting offspring’s health and development.

## Supplementary information

Supplementary results

## Data Availability

The Public Access to Information and Secrecy Act in Sweden prohibits us from making individual level data publicly available. Researchers who are interested in replicating our work can apply for individual level data from the National Board of Health and Welfare (https://www.socialstyrelsen.se/en/statistics-and-data/registers/).

## Patient and public involvement statement

Patients or the public were not involved in the design, conduct, or reporting, or dissemination of our research.
