# Supplementary material for: Association between parental ADHD and adverse offspring outcomes: Evidence from a nationwide Swedish register-based study
Source: Mol Psychiatry. 2026 Jul 27;31(12):7178–85. doi: 10.1038/s41380-026-03770-6 (PMC13736513; doi:10.1038/s41380-026-03770-6)
Supplement: Supplementary file 1 [file 41380_2026_3770_MOESM1_ESM.pdf]

Association between parental ADHD and adverse offspring outcomes

Contents

Supplementary Table 1. The definition of outcomes and covariates 2

Supplementary Table 2. Summary of baseline characteristics stratified by parental ADHD status. 4

Supplementary Table 3. Absolute rates of adverse outcomes in offspring with/without parental ADHD, stratified by parental ADHD status (per 1,000 person-years). 6

Supplementary Table 4. Number and proportion of offspring with ADHD diagnosed preceding each outcome among individuals with that outcome. 8

Supplementary Table 5. Associations between parental ADHD and offspring adverse outcomes by parental ADHD status after additional adjustment for offspring lifetime ADHD. 9

Supplementary Table 6. Sensitivity analysis: Associations between parental ADHD and offspring adverse outcomes after redefining parental ADHD based on diagnosis by parental ADHD status........ 10

Supplementary Table 7. Sensitivity analysis: Associations between parental ADHD and offspring adverse outcomes after restricting parents born after 1985, by parental ADHD status. 11

Supplementary Table 8. Sensitivity analysis: Associations between parental ADHD and offspring adverse outcomes after redefining ADHD exposure based on post-birth diagnosis or prescription, by parental ADHD status. 12

Supplementary Table 9. Sensitivity analysis: Associations between parental ADHD and adverse outcomes in offspring after redefining ADHD exposure as pre-birth diagnoses or prescriptions only, by parental ADHD status. 13

Supplementary Figure 1. Subgroup analysis: Associations between parental ADHD (at least one parent) and adverse offspring outcomes, by offspring sex. 14

Supplementary Figure 2. Subgroup analysis: Associations between maternal ADHD and adverse offspring outcomes, by offspring sex. 15

Supplementary Figure 3. Subgroup analysis: Associations between paternal ADHD and adverse offspring outcomes, by offspring sex. 16

Supplementary Figure 4. Subgroup analysis: Associations between parental ADHD (both parents) and adverse offspring outcomes, by offspring sex. 17

Supplementary Figure 5. Subgroup analysis: Associations between any parental ADHD and adverse offspring outcomes, stratified by maternal birth year (before 1980 vs. 1980 or later). 18

Supplementary Figure 6. Subgroup analysis: Associations between any parental ADHD and adverse offspring outcomes, stratified by paternal birth year (before 1980 vs. 1980 or later). 19

Supplementary Table 1. The definition of outcomes and covariates

	ICD-8	ICD-9	ICD-10	ATC code
ADHD		314	F90	N06BA01, N06BA0, N06BA04, N06BA09, N06BA12,C02AC02
Autism		299A	F845, F840	
Conduct disorder		312	F91	
Intellectual disability	311,312, 313, 314, 315	317, 318, 319	F70, F71, F72, F73, F74, F75, F76, F77, F78, F79	
Tic disorder	3062	307C	F950, F951, F952, F958, F959	
Anxiety	3000, 3001, 3002, 3005, 3006, 3007, 3008, 3009	300A, 300B, 300C, 300F, 300G, 300H, 300I	F40, F41, F44, F45, F48	
Major depressive disorder	2980, 2962, 3004	296B, 296X, 298A, 300E	F32, F33	
Accidental injuries	E8, E90, E91, E92, E93, E94	E8, E90, E91, E92, E94	V**, W**, X00–X59	
Assault/victimization	E96	E96	X85–X90, Y00–Y09	
Falls	E88	E88	W00–W19	
Intentional self-harm	E95, E98	E95, E98	X60–X84, Y10–Y34	
Poisoning	E85, E86, E87	E85, E86	X40–X49	
Suicide behavior	950–959, 980–989	950–959, 980–989	X60–X84, Y10–Y34, Y870, Y872	
Transport injuries	E80, E81, E82, E83, E84	E80, E81, E82, E83, E84	V01–V99	
Traumatic brain injury	800–804, 850–854	800–804, 850–854	S02.0–S02.9, S04.0, S06.*, S07.1	
Asthma	493	493	J45, J46	
Any autoimmune diseases	0341, 13607, 24200, 24503, 25810, 26910, 28700, 28710, 340, 35401, 390, 391, 392, 44609, 44630, 44638, 44640, 56300, 56310, 57190, 580, 582, 694, 69610, 69619, 69620, 69621, 69622, 69623, 70400, 71200, 71210, 71239, 71600, 73300, 73400, 73410	034B, 136B, 242A, 245C, 258B, 287A, 287D, 340, 357A, 358A, 390, 391, 392, 446A, 446B, 446F, 446G, 555, 556, 571F, 579A, 580, 582, 694A, 694E, 694F, 696, 704A, 710A–710D, 710W, 714A, 725	A389, D686, D690, D693, E050, E063, E310, G04, G131, G35, G610, G700, I00, I01, I02, L100, L120, L13, L40, L63, K900, K50, K51, K743, M06, M300, M301, M303, M311, M315, M317, M32, M339, M34, M350, M351, M352, M353, N00, N01, N03, N05	
Bacterial infection		001–005, 008A–008F, 01–02, 030–041, 073, 076, 077A, 078D, 078J, 080–084, 087, 091–099C, 100–104, 320–326, 382, 390–391, 475, 481–482, 510,	A00–A05, A1–A7, B95–B96, G00–G09, H66, H670, I00–I01, J13–J15, J170–J202, J36, J390–J391, J85–J86, K35–K37, L0, M00–M013, N10–N12, N30, N340, N39	

		513, 540–542, 590, 595, 597A, 599A, 614–616		
Epilepsy	345	345	G40, G41	
Gastrointestinal autoimmune diseases	26910, 56300, 56310	555, 556, 579A	K50 K51 K900	
Obesity	277	278A, 278B	E65, E66	
Sleep disorder	30640	307E, 780F	G47	
Type 1 diabetes	250	250	E10	
Viral infection		008H–008W, 045–049, 05–06, 070–072, 074–075, 077B–077X, 078A–078H, 078W, 079, 321E, 321H, 480, 487, 711F, 790W	A08, A8, A9, B0–B2, B30, B33–B34, B97, G020, G051, H671, J10–J12, J171, J203–J207, J210, M014–M015	
**Covariates**				
Substance use disorder		291, 292, 303, 304	F10, F11, F12, F13, F14, F15, F16, F18, F19	
Bipolar disorder	296	296	F30, F31	
Obsessive-compulsive disorder	3003	300D	F42	
Personality disorder	301	301	F6	
PTSD	3079	309B	F431	
Schizophrenia	2950, 2951, 2952, 2953, 2954, 2956, 2958, 2959	295A, 295B, 295C, 295D, 295E, 295G, 295W, 295X	F20	

Supplementary Table 2. Summary of baseline characteristics stratified by parental ADHD status.

	After matching					
	Maternal ADHD		Paternal ADHD		Both parents with ADHD	
	ADHD	Non-ADHD	ADHD	Non-ADHD	ADHD	Non-ADHD
**Children characteristics**						
Sex						
Males	16,755 (52.7%)	160,212 (52.7%)	15,359 (52.3%)	147,301 (52.3%)	2,278 (52.4%)	21,345 (52.3%)
Females	15,054 (47.3%)	143,741 (47.3%)	14,035 (47.8%)	134,279 (47.7%)	2,073 (47.6%)	19,472 (47.7%)
Birthday year						
2000 - 2006	12,724 (40.0%)	121,037 (39.8%)	10,870 (37.0%)	103,736 (36.8%)	1,380 (31.7%)	12,866 (31.5%)
2007 - 2014	19,085 (60.0%)	182,916 (60.2%)	18,524 (63.0%)	177,844 (63.2%)	2,971 (68.3%)	27,951 (68.5%)
**Mother characteristics**						
Birthday year						
Before 1950	0 (0.0%)	0 (0.0%)	0 (0.0%)	0 (0.0%)	0 (0.0%)	0 (0.0%)
1950 – 1960	55 (0.2%)	314 (0.1%)	46 (0.2%)	296 (0.1%)	0 (0.0%)	0 (0.0%)
1961 - 1970	3,107 (9.8%)	29,353 (9.7%)	2,666 (9.1%)	25,126 (8.9%)	261 (6.0%)	2,403 (5.9%)
1971 - 1980	13,774 (43.3%)	134,031 (44.1%)	12,207 (41.5%)	119,335 (42.4%)	1,466 (33.7%)	14,121 (34.6%)
After 1980	14,873 (46.8%)	140,255 (46.1%)	14,475 (49.2%)	136,823 (48.6%)	2,624 (60.3%)	24,293 (59.5%)
Education levels						
Primary and lower secondary education	10,130 (31.9%)	42,767 (14.1%)	7,466 (25.4%)	40,208 (14.3%)	2,009 (46.2%)	7,584 (18.6%)
Upper secondary education	14,972 (47.1%)	139,401 (45.9%)	14,292 (48.6%)	128,148 (45.5%)	1,781 (40.9%)	19,116 (46.8%)
Post-secondary and postgraduate education	6,220 (19.6%)	113,918 (37.5%)	7,221 (24.6%)	105,714 (37.5%)	453 (10.4%)	12,678 (31.1%)
Unknown	487 (1.5%)	7,867 (2.6%)	415 (1.4%)	7,510 (2.7%)	108 (2.5%)	1,439 (3.6%)
Any psychiatric comorbidity before children born	11,465 (36.0%)	20,941 (6.9%)	5,874 (20.0%)	20,221 (7.1%)	2,088 (48.0%)	3,337 (8.7%)
**Father characteristics**						
Birthday year						
Before 1950	22 (0.1%)	32 (0.0%)	2 (0.0%)	3 (0.0%)	1 (0.0%)	1 (0.0%)
1950 – 1960	646 (2.0%)	4,616 (1.5%)	403 (1.4%)	3,060 (1.1%)	58 (1.3%)	441 (1.1%)
1961 - 1970	5,485 (17.2%)	52,278 (17.2%)	4,440 (15.1%)	42,434 (15.1%)	510 (11.7%)	4,747 (11.6%)
1971 - 1980	14,825 (46.6%)	145,176 (47.8%)	13,672 (46.5%)	134,066 (47.6%)	1,758 (40.4%)	17,009 (41.7%)
After 1980	10,831 (34.1%)	101,851 (33.5%)	10,877 (37.0%)	102,017 (36.2%)	2,024 (46.5%)	18,619 (45.6%)
Education levels						
Primary and lower secondary education	7,173 (22.6%)	44,115 (14.5%)	10,129 (34.5%)	41,276 (14.7%)	1,925 (44.2%)	7,268 (17.8%)
Upper secondary education	18,244 (57.4%)	163,309 (53.7%)	14,652 (49.9%)	150,799 (53.6%)	2,034 (46.8%)	22,275 (54.6%)
Post-secondary and postgraduate education	5,963 (18.8%)	90,644 (29.8%)	4,261 (14.5%)	83,910 (29.8%)	331 (7.6%)	10,270 (25.2%)
Unknown	429 (1.3%)	5,885 (1.9%)	352 (1.2%)	5,595 (2.0%)	61 (1.4%)	1,004 (2.5%)

Any psychiatric comorbidity before children born	4,643 (14.6%)	13,000 (4.3%)	9,121 (31.0%)	12,280 (4.4%)	1,890 (43.4%)	2,054 (5.0%)

Supplementary Table 3. Absolute rates of adverse outcomes in offspring with/without parental ADHD, stratified by parental ADHD status (per 1,000 person-years).

	Maternal ADHD		Paternal ADHD		Both parents with ADHD	
	ADHD	Non-ADHD	ADHD	Non-ADHD	ADHD	Non-ADHD
**Psychiatric disorders**						
ADHD	24.61 (24.10-25.12)	4.71 (4.64-4.78)	20.89 (20.41-21.39)	4.70 (4.63-4.77)	35.21 (33.50-37.00)	5.03 (4.83-5.23)
Autism	7.45 (7.18-7.72)	1.82 (1.78-1.87)	5.83 (5.58-6.09)	1.84 (1.79-1.88)	10.32 (9.45-11.25)	1.87 (1.75-2.00)
Conduct disorder	2.28 (2.13-2.43)	0.48 (0.46-0.50)	2.12 (1.97-2.27)	0.48 (0.45-0.50)	3.80 (3.28-4.37)	0.55 (0.48-0.62)
Intellectual disability	1.30 (1.19-1.42)	0.68 (0.66-0.71)	1.43 (1.31-1.56)	0.68 (0.65-0.71)	1.78 (1.44-2.19)	0.77 (0.69-0.85)
Tic disorder	1.53 (1.42-1.66)	0.46 (0.44-0.48)	1.28 (1.16-1.40)	0.45 (0.43-0.47)	1.86 (1.51-2.27)	0.48 (0.42-0.55)
Anxiety	6.63 (6.38-6.89)	2.36 (2.31-2.41)	5.65 (5.41-5.90)	2.27 (2.22-2.32)	6.99 (6.28-7.76)	2.03 (1.91-2.16)
Major depressive disorder	4.17 (3.98-4.38)	1.54 (1.50-1.58)	3.41 (3.22-3.61)	1.51 (1.47-1.55)	3.95 (3.42-4.53)	1.33 (1.23-1.43)
**Behavioral outcomes**						
Accidental injuries	66.88 (65.90-67.87)	56.35 (56.07-56.64)	65.61 (64.59-66.64)	57.02 (56.72-57.32)	73.33 (70.45-76.28)	58.44 (57.63-59.26)
Assault/victimization	1.21 (1.10-1.32)	0.46 (0.44-0.48)	1.09 (0.99-1.21)	0.45 (0.42-0.47)	1.82 (1.47-2.22)	0.44 (0.38-0.50)
Falls	36.18 (35.52-36.84)	32.48 (32.28-32.68)	36.24 (35.55-36.94)	32.87 (32.65-33.08)	39.23 (37.33-41.21)	33.27 (32.70-33.85)
Intentional self-harm	1.86 (1.73-2.00)	1.12 (1.08-1.15)	1.93 (1.79-2.08)	1.09 (1.06-1.13)	2.32 (1.92-2.77)	1.10 (1.01-1.20)
Poisoning	1.34 (1.23-1.45)	0.60 (0.58-0.63)	1.13 (1.02-1.24)	0.62 (0.59-0.64)	2.09 (1.71-2.53)	0.71 (0.63-0.79)
Suicide behavior	1.87 (1.74-2.01)	1.12 (1.09-1.16)	1.95 (1.81-2.10)	1.10 (1.06-1.13)	2.34 (1.94-2.79)	1.11 (1.01-1.20)
Transport injuries	6.43 (6.18-6.68)	5.68 (5.60-5.76)	6.80 (6.53-7.07)	5.55 (5.47-5.63)	6.88 (6.17-7.65)	5.75 (5.54-5.98)
Traumatic brain injury	6.03 (5.78-6.27)	4.45 (4.38-4.51)	5.68 (5.44-5.94)	4.48 (4.40-4.55)	6.79 (6.08-7.56)	4.66 (4.47-4.86)
**Somatic diseases**						
Asthma	20.45 (19.97-20.94)	12.78 (12.66-12.90)	17.41 (16.95-17.88)	12.97 (12.84-13.10)	23.43 (22.00-24.92)	13.87 (13.52-14.23)
Any autoimmune diseases	2.75 (2.59-2.92)	2.19 (2.14-2.24)	2.49 (2.33-2.66)	2.25 (2.20-2.30)	2.55 (2.13-3.02)	2.20 (2.07-2.33)
Bacterial infection	32.87 (32.22-33.52)	23.86 (23.69-24.03)	28.74 (28.12-29.37)	24.15 (23.97-24.33)	32.73 (30.97-34.56)	24.75 (24.26-25.25)
Epilepsy	1.17 (1.06-1.28)	0.79 (0.76-0.82)	1.15 (1.04-1.26)	0.78 (0.75-0.81)	1.34 (1.04-1.69)	0.79 (0.72-0.88)
Gastrointestinal autoimmune diseases	1.24 (1.13-1.35)	1.00 (0.97-1.03)	1.05 (0.95-1.16)	1.00 (0.96-1.03)	1.12 (0.85-1.45)	0.95 (0.86-1.04)
Obesity	3.54 (3.36-3.73)	1.77 (1.73-1.81)	2.98 (2.80-3.16)	1.79 (1.74-1.83)	3.54 (3.04-4.10)	1.94 (1.82-2.07)

Sleep disorder	4.83 (4.61-5.05)	1.54 (1.50-1.58)	3.99 (3.79-4.21)	1.52 (1.48-1.56)	6.11 (5.45-6.83)	1.63 (1.52-1.75)
Type 1 diabetes	0.54 (0.47-0.61)	0.48 (0.46-0.51)	0.49 (0.42-0.56)	0.49 (0.46-0.51)	0.66 (0.45-0.92)	0.46 (0.40-0.53)
Viral infection	37.13 (36.43-37.84)	25.21 (25.03-25.39)	31.90 (31.24-32.57)	25.85 (25.66-26.04)	40.66 (38.63-42.76)	27.91 (27.38-28.44)

Abbreviations: ADHD, Attention-Deficit/Hyperactivity Disorder.

Supplementary Table 4. Number and proportion of offspring with ADHD diagnosed preceding each outcome among individuals with that outcome.

	Any parents with ADHD	
	**1**	**0**
**Psychiatric disorders**		
Autism	1702 (37.7%)	2698 (21.5%)
Conduct disorder	505 (33.7%)	772 (23.5%)
Intellectual disability	288 (30.2%)	692 (14.9%)
Tic disorder	389 (39.3%)	591 (19.1%)
Anxiety	1565 (36.0%)	2573 (16.2%)
Major depressive disorder	1081 (39.6%)	1888 (17.8%)
**Behavioral outcomes**		
Accidental injuries	1626 (5.2%)	2985 (1.1%)
Assault/victimization	294 (36.8%)	551 (17.7%)
Falls	1212 (6.0%)	2180 (1.2%)
Intentional self-harm	351 (26.3%)	644 (8.5%)
Poisoning	83 (9.9%)	129 (3.1%)
Suicide behavior	354 (26.3%)	648 (8.5%)
Transport injuries	786 (17.1%)	1498 (4.0%)
Traumatic brain injury	353 (8.8%)	579 (2.0%)
**Somatic diseases**		
Asthma	270 (2.4%)	373 (0.5%)
Any autoimmune diseases	147 (7.9%)	234 (1.6%)
Bacterial infection	539 (3.2%)	949 (0.7%)
Epilepsy	106 (13.0%)	220 (4.1%)
Gastrointestinal autoimmune diseases	65 (7.9%)	102 (1.5%)
Obesity	396 (17.1%)	751 (6.2%)
Sleep disorder	892 (29.2%)	1387 (13.4%)
Type 1 diabetes	42 (11.6%)	72 (2.2%)
Viral infection	236 (1.3%)	400 (0.3%)

Abbreviations: ADHD, Attention-Deficit/Hyperactivity Disorder.

Supplementary Table 5. Associations between parental ADHD and offspring adverse outcomes by parental ADHD status after additional adjustment for offspring lifetime ADHD.

	Maternal ADHD	Paternal ADHD	Both parents with ADHD
	**Adjusted hazard ratio (95% CI)***	**Adjusted hazard ratio (95% CI)***	**Adjusted hazard ratio (95% CI)***
**Psychiatric disorders**			
Autism	1.61 (1.51-1.71)	1.44 (1.34-1.54)	1.83 (1.56-2.14)
Conduct disorder	1.29 (1.15-1.44)	1.38 (1.22-1.56)	1.43 (1.12-1.83)
Intellectual disability	0.80 (0.71-0.90)	1.06 (0.94-1.19)	0.81 (0.61-1.08)
Tic disorder	1.27 (1.13-1.42)	1.28 (1.13-1.46)	1.30 (0.95-1.76)
Anxiety	1.49 (1.41-1.57)	1.42 (1.34-1.51)	1.74 (1.48-2.04)
Major depressive disorder	1.38 (1.29-1.48)	1.23 (1.14-1.33)	1.29 (1.04-1.60)
**Behavioral outcomes**			
Accidental injuries	1.11 (1.09-1.13)	1.08 (1.06-1.10)	1.11 (1.05-1.17)
Assault/victimization	1.43 (1.27-1.61)	1.37 (1.20-1.56)	1.71 (1.27-2.30)
Falls	1.05 (1.03-1.08)	1.06 (1.03-1.08)	1.05 (0.98-1.12)
Intentional self-harm	1.14 (1.04-1.25)	1.28 (1.17-1.40)	1.43 (1.11-1.83)
Poisoning	1.46 (1.30-1.63)	1.34 (1.18-1.51)	2.10 (1.60-2.75)
Suicide behavior	1.14 (1.04-1.25)	1.29 (1.18-1.42)	1.44 (1.12-1.84)
Transport injuries	1.00 (0.95-1.05)	1.09 (1.04-1.14)	0.95 (0.83-1.09)
Traumatic brain injury	1.19 (1.13-1.25)	1.12 (1.07-1.18)	1.13 (0.99-1.30)
**Somatic diseases**			
Asthma	1.34 (1.30-1.38)	1.16 (1.12-1.20)	1.33 (1.22-1.45)
Any autoimmune diseases	1.23 (1.15-1.32)	1.09 (1.01-1.18)	1.28 (1.02-1.59)
Bacterial infection	1.21 (1.18-1.24)	1.06 (1.04-1.09)	1.11 (1.03-1.20)
Epilepsy	0.98 (0.87-1.10)	1.13 (1.00-1.28)	0.96 (0.69-1.32)
Gastrointestinal autoimmune diseases	1.21 (1.09-1.35)	1.05 (0.93-1.18)	1.30 (0.94-1.81)
Obesity	1.33 (1.24-1.42)	1.11 (1.03-1.20)	0.95 (0.78-1.17)
Sleep disorder	1.59 (1.49-1.69)	1.44 (1.34-1.55)	1.69 (1.41-2.01)
Type 1 diabetes	1.10 (0.93-1.29)	0.99 (0.83-1.17)	1.65 (1.04-2.61)
Viral infection	1.24 (1.21-1.27)	1.09 (1.07-1.12)	1.19 (1.11-1.27)

Abbreviations: ADHD, Attention-Deficit/Hyperactivity Disorder.

Notation: Accidental injuries, falls, poisoning, suicide behavior, and transport injuries include both diagnoses and deaths; all other outcomes are based on diagnoses only. Models are adjusted for maternal and paternal education levels, offspring lifetime ADHD, as well as maternal and paternal psychiatric comorbidities. The reference group consists of offspring whose parents both do not have ADHD.

Supplementary Table 6. Sensitivity analysis: Associations between parental ADHD and offspring adverse outcomes after redefining parental ADHD based on diagnosis by parental ADHD status.

	Any parents with ADHD	Maternal ADHD	Paternal ADHD	Both parents with ADHD
	**Adjusted hazard ratio (95% CI)***	**Adjusted hazard ratio (95% CI)***	**Adjusted hazard ratio (95% CI)***	**Adjusted hazard ratio (95% CI)***
**Psychiatric disorders**				
ADHD	4.55 (4.44-4.68)	5.04 (4.88-5.21)	4.33 (4.18-4.49)	6.81 (6.21-7.46)
Autism	3.20 (3.07-3.34)	3.63 (3.44-3.83)	3.00 (2.82-3.19)	4.93 (4.26-5.71)
Conduct disorder	3.52 (3.25-3.80)	3.72 (3.38-4.09)	3.49 (3.13-3.89)	4.73 (3.72-6.03)
Intellectual disability	1.56 (1.43-1.70)	1.41 (1.26-1.59)	1.77 (1.58-1.98)	1.65 (1.24-2.20)
Tic disorder	2.79 (2.55-3.05)	3.04 (2.72-3.40)	2.64 (2.33-2.99)	3.61 (2.60-5.01)
Anxiety	2.54 (2.44-2.65)	2.76 (2.62-2.90)	2.39 (2.26-2.53)	3.34 (2.85-3.92)
Major depressive disorder	2.34 (2.23-2.47)	2.63 (2.46-2.80)	2.12 (1.97-2.28)	2.89 (2.35-3.55)
**Behavioral outcomes**				
Accidental injuries	1.12 (1.10-1.13)	1.14 (1.12-1.16)	1.10 (1.08-1.12)	1.17 (1.11-1.23)
Assault/victimization	1.79 (1.63-1.97)	1.87 (1.66-2.11)	1.80 (1.58-2.06)	2.51 (1.84-3.42)
Falls	1.06 (1.04-1.08)	1.07 (1.05-1.10)	1.06 (1.03-1.09)	1.07 (1.00-1.14)
Intentional self-harm	1.52 (1.42-1.63)	1.48 (1.35-1.61)	1.60 (1.46-1.75)	1.90 (1.47-2.46)
Poisoning	1.64 (1.50-1.79)	1.77 (1.58-1.98)	1.66 (1.47-1.88)	2.83 (2.16-3.70)
Suicide behavior	1.52 (1.42-1.63)	1.47 (1.35-1.61)	1.60 (1.45-1.75)	1.89 (1.46-2.44)
Transport injuries	1.11 (1.07-1.15)	1.07 (1.02-1.12)	1.14 (1.09-1.20)	0.98 (0.85-1.13)
Traumatic brain injury	1.22 (1.17-1.27)	1.26 (1.20-1.33)	1.18 (1.12-1.25)	1.24 (1.08-1.43)
**Somatic diseases**				
Asthma	1.34 (1.31-1.37)	1.46 (1.41-1.50)	1.25 (1.20-1.29)	1.53 (1.39-1.67)
Any autoimmune diseases	1.19 (1.12-1.26)	1.26 (1.17-1.36)	1.10 (1.01-1.19)	1.20 (0.95-1.53)
Bacterial infection	1.18 (1.16-1.20)	1.26 (1.22-1.29)	1.10 (1.07-1.13)	1.18 (1.09-1.27)
Epilepsy	1.32 (1.21-1.45)	1.27 (1.13-1.42)	1.39 (1.23-1.58)	1.33 (0.94-1.88)
Gastrointestinal autoimmune diseases	1.17 (1.07-1.27)	1.27 (1.14-1.41)	1.06 (0.93-1.20)	1.28 (0.89-1.83)
Obesity	1.54 (1.46-1.62)	1.65 (1.54-1.77)	1.39 (1.28-1.50)	1.35 (1.09-1.66)
Sleep disorder	2.50 (2.38-2.63)	2.78 (2.62-2.96)	2.33 (2.17-2.50)	3.70 (3.11-4.40)
Type 1 diabetes	1.06 (0.94-1.21)	1.11 (0.94-1.31)	1.06 (0.89-1.27)	1.36 (0.83-2.23)
Viral infection	1.21 (1.19-1.24)	1.30 (1.27-1.33)	1.13 (1.10-1.16)	1.23 (1.14-1.32)

Abbreviations: ADHD, Attention-Deficit/Hyperactivity Disorder.

Notation: Accidental injuries, falls, poisoning, suicide behavior, and transport injuries include both diagnoses and deaths; all other outcomes are based on diagnoses only. All models are adjusted for maternal and paternal education levels, as well as maternal and paternal psychiatric comorbidities. The reference group consists of offspring with no parental ADHD. After matching, 50,321 offspring with any parental ADHD were matched to 481,504 individuals without parental ADHD; 28,147 with maternal ADHD were matched to 268,586 individuals without parental ADHD; 25,872 with paternal ADHD were matched to 247,564 individuals without parental ADHD; and 3,698 with both parents having ADHD were matched to 34,646 individuals without parental ADHD.

Supplementary Table 7. Sensitivity analysis: Associations between parental ADHD and offspring adverse outcomes after restricting parents born after 1985, by parental ADHD status.

	Any parents with ADHD	Maternal ADHD	Paternal ADHD	Both parents with ADHD
	Adjusted hazard ratio (95% CI)*	Adjusted hazard ratio (95% CI)*	Adjusted hazard ratio (95% CI)*	Adjusted hazard ratio (95% CI)*
**Psychiatric disorders**				
ADHD	3.39 (3.18-3.62)	3.77 (3.49-4.08)	3.60 (3.24-4.00)	6.27 (5.05-7.79)
Autism	2.01 (1.81-2.24)	2.33 (2.04-2.66)	2.17 (1.82-2.60)	3.53 (2.48-5.04)
Conduct disorder	2.98 (2.52-3.52)	2.93 (2.41-3.57)	3.58 (2.72-4.71)	7.46 (4.44-12.53)
Intellectual disability	1.15 (0.94-1.40)	1.04 (0.80-1.35)	1.54 (1.16-2.06)	1.87 (1.09-3.20)
Tic disorder	2.94 (2.40-3.60)	2.68 (2.07-3.46)	3.82 (2.72-5.36)	13.88 (6.17-31.24)
Anxiety	2.01 (1.73-2.33)	2.24 (1.86-2.69)	1.75 (1.31-2.34)	3.33 (1.90-5.83)
Major depressive disorder	1.88 (1.51-2.34)	2.06 (1.59-2.68)	1.95 (1.22-3.10)	3.14 (1.25-7.90)
**Behavioral outcomes**				
Accidental injuries	1.13 (1.10-1.16)	1.17 (1.13-1.21)	1.10 (1.05-1.15)	1.17 (1.05-1.30)
Assault/victimization	1.63 (1.25-2.13)	1.68 (1.21-2.32)	1.72 (1.07-2.76)	2.40 (1.04-5.55)
Falls	1.11 (1.08-1.15)	1.13 (1.08-1.19)	1.07 (1.01-1.13)	1.07 (0.94-1.22)
Intentional self-harm	1.43 (1.20-1.71)	1.40 (1.10-1.77)	1.33 (0.97-1.81)	2.52 (1.01-6.24)
Poisoning	1.45 (1.20-1.75)	1.55 (1.25-1.93)	1.33 (0.99-1.80)	2.36 (1.40-3.96)
Suicide behavior	1.44 (1.21-1.72)	1.39 (1.10-1.77)	1.36 (1.00-1.85)	2.50 (1.02-6.11)
Transport injuries	1.05 (0.96-1.14)	1.05 (0.94-1.18)	1.17 (1.01-1.35)	1.15 (0.82-1.62)
Traumatic brain injury	1.22 (1.12-1.32)	1.26 (1.13-1.40)	1.11 (0.97-1.28)	1.33 (0.99-1.79)
**Somatic diseases**				
Asthma	1.32 (1.26-1.38)	1.45 (1.37-1.54)	1.16 (1.08-1.26)	1.42 (1.19-1.68)
Any autoimmune diseases	1.08 (0.95-1.23)	1.14 (0.96-1.35)	1.06 (0.85-1.32)	0.92 (0.50-1.70)
Bacterial infection	1.08 (1.04-1.13)	1.17 (1.11-1.23)	0.99 (0.93-1.06)	1.18 (1.02-1.38)
Epilepsy	1.34 (1.11-1.60)	1.38 (1.10-1.73)	1.39 (1.02-1.90)	1.50 (0.77-2.90)
Gastrointestinal autoimmune diseases	1.10 (0.90-1.35)	1.29 (1.00-1.66)	0.70 (0.45-1.07)	0.56 (0.15-2.12)
Obesity	1.22 (1.08-1.37)	1.26 (1.09-1.47)	1.02 (0.83-1.26)	1.09 (0.69-1.71)
Sleep disorder	2.03 (1.82-2.27)	2.29 (2.02-2.60)	2.05 (1.70-2.46)	3.30 (2.29-4.75)
Type 1 diabetes	1.00 (0.74-1.35)	1.14 (0.77-1.69)	1.55 (0.93-2.57)	2.96 (0.91-9.63)
Viral infection	1.11 (1.07-1.15)	1.19 (1.14-1.24)	1.04 (0.98-1.10)	1.15 (1.02-1.31)

Abbreviations: ADHD, Attention-Deficit/Hyperactivity Disorder.

Notation: Accidental injuries, falls, poisoning, suicide behavior, and transport injuries include both diagnoses and deaths; all other outcomes are based on diagnoses only. All models are adjusted for maternal and paternal education levels, as well as maternal and paternal psychiatric comorbidities. The reference group consists of offspring with no parental ADHD. After matching, 15,602 offspring with any parental ADHD were matched to 145,844 individuals without parental ADHD; 8,780 with maternal ADHD were matched to 81,930 individuals without parental ADHD; 5,945 with paternal ADHD were matched to 54,520 individuals without parental ADHD; and 1,051 with both parents having ADHD were matched to 9,618 individuals without parental ADHD.

Supplementary Table 8. Sensitivity analysis: Associations between parental ADHD and offspring adverse outcomes after redefining ADHD exposure based on post-birth diagnosis or prescription, by parental ADHD status.

	**Any parents with ADHD**	**Maternal ADHD**	**Paternal ADHD**	**Both parents with ADHD**
	**Adjusted hazard ratio (95% CI)***	**Adjusted hazard ratio (95% CI)***	**Adjusted hazard ratio (95% CI)***	**Adjusted hazard ratio (95% CI)***
**Psychiatric disorders**				
ADHD	4.49 (4.38-4.60)	4.98 (4.83-5.14)	4.30 (4.16-4.45)	7.24 (6.65-7.88)
Autism	3.17 (3.04-3.30)	3.58 (3.41-3.77)	2.96 (2.80-3.14)	5.15 (4.49-5.91)
Conduct disorder	3.32 (3.08-3.57)	3.55 (3.24-3.88)	3.29 (2.97-3.64)	4.99 (3.96-6.27)
Intellectual disability	1.48 (1.37-1.61)	1.38 (1.23-1.53)	1.61 (1.44-1.80)	1.46 (1.10-1.94)
Tic disorder	2.80 (2.58-3.03)	3.03 (2.73-3.35)	2.69 (2.39-3.02)	4.38 (3.20-5.99)
Anxiety	2.52 (2.43-2.62)	2.74 (2.61-2.87)	2.37 (2.25-2.50)	3.23 (2.80-3.72)
Major depressive disorder	2.27 (2.16-2.38)	2.53 (2.39-2.69)	2.04 (1.91-2.19)	2.67 (2.23-3.20)
**Behavioral outcomes**				
Accidental injuries	1.13 (1.11-1.14)	1.14 (1.12-1.16)	1.12 (1.10-1.14)	1.16 (1.10-1.22)
Assault/victimization	1.79 (1.64-1.95)	1.86 (1.66-2.08)	1.84 (1.63-2.07)	2.77 (2.07-3.72)
Falls	1.07 (1.05-1.09)	1.07 (1.05-1.10)	1.07 (1.05-1.10)	1.07 (1.00-1.14)
Intentional self-harm	1.50 (1.41-1.60)	1.48 (1.36-1.61)	1.60 (1.47-1.75)	1.95 (1.54-2.47)
Poisoning	1.62 (1.49-1.76)	1.78 (1.60-1.97)	1.58 (1.41-1.78)	2.83 (2.22-3.60)
Suicide behavior	1.50 (1.41-1.60)	1.47 (1.35-1.60)	1.61 (1.47-1.75)	1.93 (1.53-2.44)
Transport injuries	1.09 (1.06-1.13)	1.05 (1.00-1.10)	1.14 (1.09-1.20)	1.04 (0.92-1.19)
Traumatic brain injury	1.23 (1.19-1.28)	1.26 (1.21-1.33)	1.19 (1.14-1.26)	1.20 (1.05-1.37)
**Somatic diseases**				
Asthma	1.34 (1.31-1.37)	1.45 (1.41-1.49)	1.24 (1.20-1.29)	1.49 (1.37-1.63)
Any autoimmune diseases	1.20 (1.14-1.27)	1.27 (1.19-1.36)	1.14 (1.05-1.23)	1.32 (1.06-1.64)
Bacterial infection	1.20 (1.18-1.22)	1.27 (1.24-1.30)	1.13 (1.10-1.16)	1.23 (1.14-1.32)
Epilepsy	1.33 (1.22-1.45)	1.31 (1.18-1.46)	1.39 (1.23-1.56)	1.62 (1.19-2.20)
Gastrointestinal autoimmune diseases	1.18 (1.09-1.28)	1.27 (1.15-1.41)	1.11 (0.99-1.25)	1.43 (1.05-1.96)
Obesity	1.54 (1.47-1.63)	1.66 (1.56-1.77)	1.40 (1.30-1.50)	1.43 (1.19-1.73)
Sleep disorder	2.45 (2.34-2.57)	2.69 (2.54-2.86)	2.31 (2.15-2.47)	3.31 (2.79-3.92)
Type 1 diabetes	1.05 (0.93-1.18)	1.15 (0.98-1.34)	1.00 (0.84-1.18)	1.26 (0.80-1.97)
Viral infection	1.23 (1.20-1.25)	1.31 (1.28-1.34)	1.15 (1.12-1.18)	1.31 (1.22-1.40)

Abbreviations: ADHD, Attention-Deficit/Hyperactivity Disorder.

Notation: Accidental injuries, falls, poisoning, suicide behavior, and transport injuries include both diagnoses and deaths; all other outcomes are based on diagnoses only. All models are adjusted for maternal and paternal education levels, as well as maternal and paternal psychiatric comorbidities. The reference group consists of offspring with no parental ADHD. After matching, 54,220 offspring with any parental ADHD were matched to 519,881 individuals without parental ADHD; 30,472 with maternal ADHD were matched to 291,410 individuals without parental ADHD; 27,724 with paternal ADHD were matched to 265,938 individuals without parental ADHD; and 3,976 with both parents having ADHD were matched to 37,467 individuals without parental ADHD.

Supplementary Table 9. Sensitivity analysis: Associations between parental ADHD and adverse outcomes in offspring after redefining ADHD exposure as pre-birth diagnoses or prescriptions only, by parental ADHD status.

	Any parents with ADHD	Maternal ADHD	Paternal ADHD
	Adjusted hazard ratio (95% CI)*	Adjusted hazard ratio (95% CI)*	Adjusted hazard ratio (95% CI)*
**Psychiatric disorders**			
ADHD	1.98 (1.72-2.28)	1.88 (1.50-2.35)	2.10 (1.74-2.53)
Autism	1.56 (1.24-1.97)	1.22 (0.84-1.79)	1.93 (1.43-2.61)
Conduct disorder	1.52 (1.03-2.23)	1.12 (0.58-2.17)	1.80 (1.10-2.96)
Intellectual disability	1.73 (1.26-2.38)	2.36 (1.43-3.89)	1.43 (0.90-2.28)
Tic disorder	2.64 (1.66-4.21)	2.24 (1.02-4.93)	2.71 (1.46-5.03)
Anxiety	1.33 (0.95-1.86)	0.94 (0.54-1.65)	1.52 (0.98-2.36)
Major depressive disorder	1.45 (0.80-2.62)	1.35 (0.48-3.81)	1.35 (0.63-2.87)
**Behavioral outcomes**			
Accidental injuries	1.13 (1.07-1.20)	1.10 (1.00-1.21)	1.17 (1.08-1.27)
Assault/victimization	1.14 (0.59-2.21)	1.34 (0.56-3.24)	0.65 (0.21-2.02)
Falls	1.17 (1.09-1.26)	1.10 (0.98-1.24)	1.24 (1.13-1.36)
Intentional self-harm	1.27 (0.85-1.89)	0.95 (0.49-1.83)	1.42 (0.84-2.38)
Poisoning	1.04 (0.70-1.56)	0.74 (0.39-1.42)	1.16 (0.69-1.96)
Suicide behavior	1.32 (0.89-1.95)	0.94 (0.49-1.80)	1.51 (0.91-2.50)
Transport injuries	1.02 (0.83-1.26)	1.04 (0.76-1.44)	1.03 (0.78-1.35)
Traumatic brain injury	1.18 (0.99-1.41)	1.02 (0.78-1.35)	1.27 (1.01-1.60)
**Somatic diseases**			
Asthma	1.22 (1.11-1.34)	1.26 (1.09-1.45)	1.17 (1.03-1.33)
Any autoimmune diseases	1.08 (0.82-1.43)	1.34 (0.92-1.96)	0.90 (0.59-1.38)
Bacterial infection	0.98 (0.90-1.07)	1.00 (0.88-1.14)	0.96 (0.86-1.08)
Epilepsy	1.62 (1.11-2.37)	1.64 (0.89-3.02)	1.45 (0.86-2.45)
Gastrointestinal autoimmune diseases	1.15 (0.74-1.78)	1.68 (0.92-3.09)	0.86 (0.45-1.65)
Obesity	0.95 (0.71-1.26)	0.88 (0.57-1.34)	0.91 (0.62-1.34)
Sleep disorder	1.41 (1.10-1.79)	1.48 (1.02-2.17)	1.40 (1.00-1.97)
Type 1 diabetes	0.80 (0.43-1.49)	0.66 (0.28-1.51)	1.19 (0.50-2.83)
Viral infection	1.10 (1.03-1.19)	1.16 (1.04-1.30)	1.08 (0.98-1.19)

Abbreviations: ADHD, Attention-Deficit/Hyperactivity Disorder.

Notation: Accidental injuries, falls, poisoning, suicide behavior, and transport injuries include both diagnoses and deaths; all other outcomes are based on diagnoses only. All models are adjusted for maternal and paternal education levels, as well as maternal and paternal psychiatric comorbidities. The reference group consists of offspring with no parental ADHD. After matching, 2,998 offspring with any parental ADHD were matched to 28,656 individuals without parental ADHD; 1,350 with maternal ADHD were matched to 12,898 individuals without parental ADHD; 1,684 with paternal ADHD were matched to 16,079 individuals without parental ADHD; and 36 with both parents having ADHD were matched to 321 individuals without parental ADHD. Due to the small sample size of individuals with both parents having ADHD, we did not conduct analyses for this subgroup.

Outcomes	Any parents with ADHD − male	Any parents with ADHD − female
**Psychiatric disorders**		
ADHD	4.22 (4.10−4.35)	4.97 (4.77−5.18)
Autism	3.00 (2.86−3.16)	3.46 (3.22−3.72)
Conduct disorder	3.32 (3.04−3.64)	3.52 (3.07−4.04)
Intellectual disability	1.52 (1.37−1.69)	1.45 (1.27−1.65)
Tic disorder	2.86 (2.60−3.14)	2.71 (2.27−3.23)
Anxiety	2.57 (2.42−2.74)	2.45 (2.33−2.57)
Major depression disorder	2.59 (2.39−2.80)	2.15 (2.03−2.28)
**Behavioral outcomes**		
Accidental injuries	1.11 (1.09−1.13)	1.14 (1.12−1.16)
Assault/victimization	1.71 (1.51−1.94)	1.95 (1.71−2.22)
Falls	1.06 (1.04−1.09)	1.09 (1.06−1.11)
Intentional self−harm	1.46 (1.32−1.60)	1.57 (1.44−1.71)
Poisoning	1.63 (1.46−1.83)	1.60 (1.40−1.82)
Suicide behavior	1.46 (1.33−1.61)	1.57 (1.44−1.71)
Transport injuries	1.04 (0.99−1.09)	1.18 (1.12−1.23)
Traumatic brain injury	1.20 (1.15−1.26)	1.25 (1.19−1.32)
**Somatic diseases**		
Asthma	1.33 (1.30−1.37)	1.34 (1.30−1.39)
Autoimmune diseases	1.24 (1.15−1.34)	1.15 (1.07−1.24)
Bacterial infection	1.20 (1.17−1.23)	1.18 (1.15−1.22)
Epilepsy	1.32 (1.18−1.47)	1.32 (1.16−1.49)
Gastrointestinal autoimmune diseases	1.34 (1.19−1.51)	1.06 (0.96−1.18)
Obesity	1.57 (1.46−1.68)	1.48 (1.38−1.59)
Sleep disorder	2.47 (2.32−2.63)	2.47 (2.30−2.65)
Type 1 diabetes	0.96 (0.81−1.14)	1.14 (0.97−1.35)
Viral infection	1.21 (1.18−1.24)	1.22 (1.19−1.26)

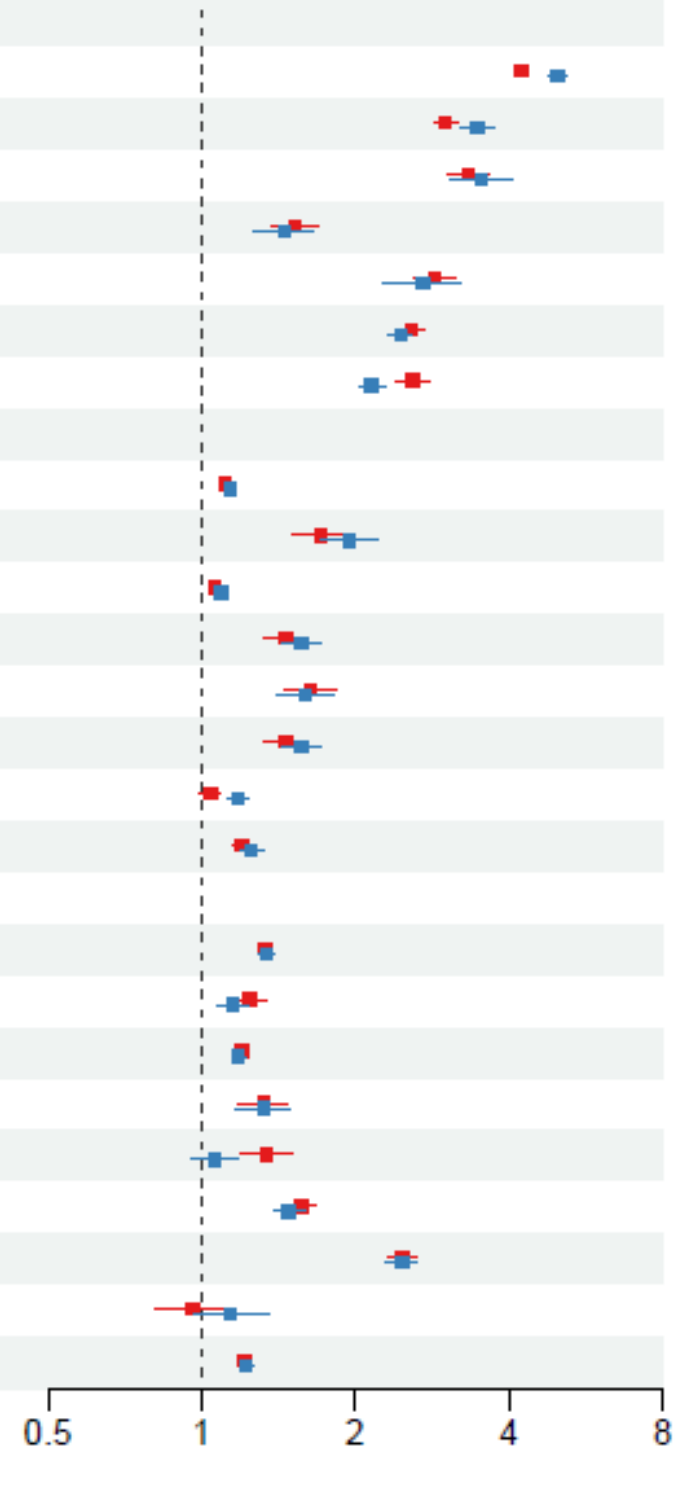

Supplementary Figure 1. Subgroup analysis: Associations between parental ADHD (at least one parent) and adverse offspring outcomes, by offspring sex.

Abbreviations: ADHD, Attention-Deficit/Hyperactivity Disorder.

Notation: Accidental injuries, falls, poisoning, suicide behavior, and transport injuries include both diagnoses and deaths; all other outcomes are based on diagnoses only. Models are adjusted for maternal and paternal education levels, as well as maternal and paternal psychiatric comorbidities. The reference group consists of offspring whose parents both do not have ADHD. After matching, 16,755 male offspring with maternal ADHD were matched to 160,212 male offspring without parental ADHD; 27,016 female offspring with any paternal ADHD were matched to 258,548 female offspring without parental ADHD.

Outcomes	Mother with ADHD – male	Mother with ADHD – female
**Psychiatric disorders**		
ADHD	4.60 (4.43–4.78)	5.69 (5.41–5.99)
Autism	3.39 (3.19–3.61)	4.03 (3.70–4.39)
Conduct disorder	3.42 (3.06–3.81)	4.10 (3.49–4.82)
Intellectual disability	1.41 (1.23–1.61)	1.36 (1.14–1.62)
Tic disorder	3.03 (2.69–3.40)	3.12 (2.50–3.90)
Anxiety	2.78 (2.58–3.01)	2.68 (2.53–2.85)
Major depression disorder	2.95 (2.68–3.26)	2.38 (2.21–2.56)
**Behavioral outcomes**		
Accidental injuries	1.13 (1.10–1.15)	1.17 (1.14–1.20)
Assault/victimization	1.92 (1.65–2.23)	1.92 (1.62–2.27)
Falls	1.06 (1.03–1.09)	1.10 (1.07–1.14)
Intentional self–harm	1.43 (1.26–1.62)	1.55 (1.38–1.74)
Poisoning	1.79 (1.57–2.06)	1.76 (1.50–2.06)
Suicide behavior	1.44 (1.27–1.63)	1.54 (1.37–1.73)
Transport injuries	1.01 (0.95–1.07)	1.12 (1.05–1.20)
Traumatic brain injury	1.26 (1.18–1.34)	1.27 (1.19–1.37)
**Somatic diseases**		
Asthma	1.44 (1.38–1.49)	1.47 (1.40–1.54)
Autoimmune diseases	1.34 (1.22–1.49)	1.19 (1.08–1.30)
Bacterial infection	1.26 (1.22–1.30)	1.27 (1.23–1.32)
Epilepsy	1.29 (1.12–1.49)	1.22 (1.04–1.43)
Gastrointestinal autoimmune diseases	1.46 (1.26–1.69)	1.14 (0.99–1.30)
Obesity	1.65 (1.50–1.80)	1.66 (1.51–1.82)
Sleep disorder	2.74 (2.53–2.96)	2.68 (2.45–2.92)
Type 1 diabetes	1.03 (0.83–1.29)	1.24 (1.00–1.54)
Viral infection	1.28 (1.24–1.32)	1.32 (1.28–1.37)

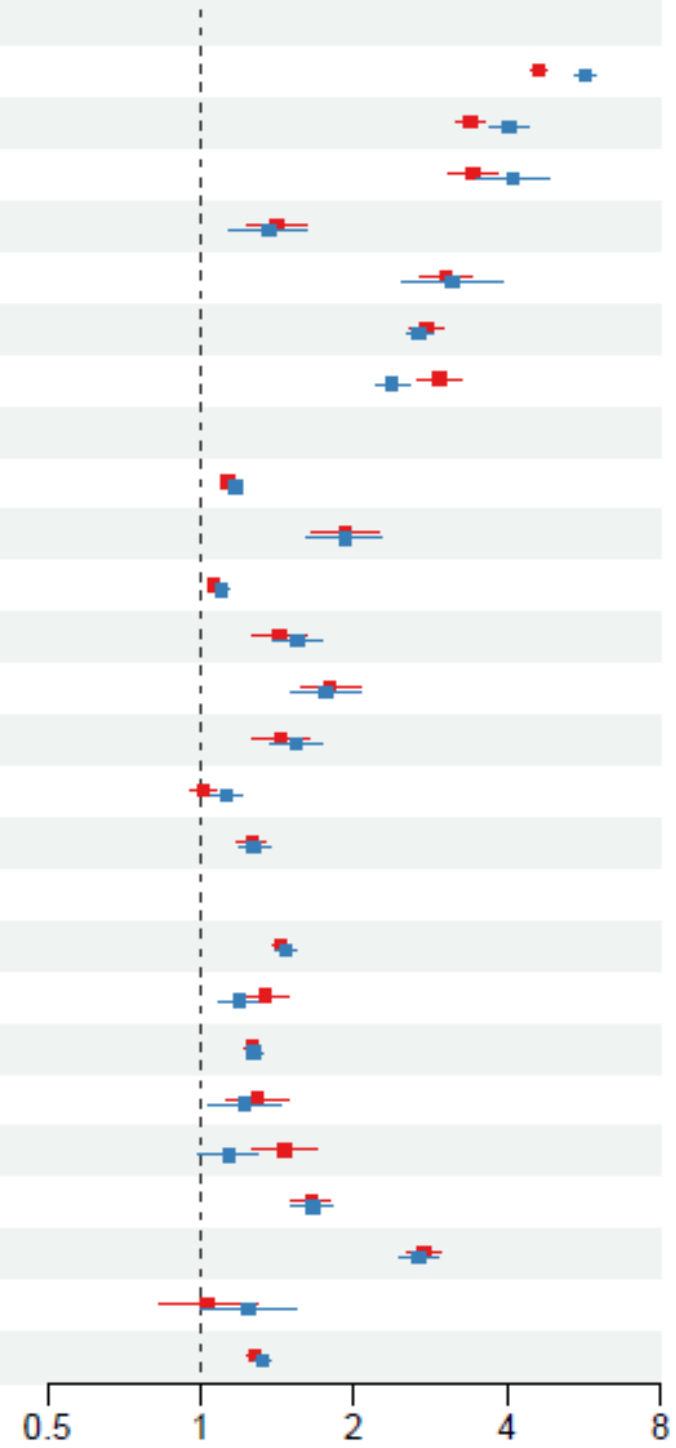

Sex of index person Male Female

Supplementary Figure 2. Subgroup analysis: Associations between maternal ADHD and adverse offspring outcomes, by offspring sex.

Abbreviations: ADHD, Attention-Deficit/Hyperactivity Disorder.

Notation: Accidental injuries, falls, poisoning, suicide behavior, and transport injuries include both diagnoses and deaths; all other outcomes are based on diagnoses only. Models are adjusted for maternal and paternal education levels, as well as maternal and paternal psychiatric comorbidities. The reference group consists of offspring whose parents both do not have ADHD. After matching, 15,359 male offspring with paternal ADHD were matched to 147,301 male offspring without parental ADHD; 15,054 female offspring with maternal ADHD were matched to 143,741 female offspring without parental ADHD.

Outcomes	Father with ADHD – male	Father with ADHD – female
**Psychiatric disorders**		
ADHD	4.12 (3.95–4.29)	4.60 (4.35–4.86)
Autism	2.85 (2.66–3.05)	3.19 (2.87–3.54)
Conduct disorder	3.36 (2.98–3.80)	3.39 (2.81–4.10)
Intellectual disability	1.75 (1.54–2.00)	1.45 (1.22–1.73)
Tic disorder	2.80 (2.45–3.20)	2.35 (1.83–3.01)
Anxiety	2.47 (2.26–2.70)	2.25 (2.10–2.41)
Major depression disorder	2.27 (2.03–2.54)	1.98 (1.82–2.16)
**Behavioral outcomes**		
Accidental injuries	1.10 (1.07–1.13)	1.12 (1.09–1.15)
Assault/victimization	1.53 (1.29–1.82)	2.25 (1.89–2.68)
Falls	1.07 (1.04–1.10)	1.08 (1.05–1.12)
Intentional self–harm	1.51 (1.33–1.72)	1.64 (1.46–1.85)
Poisoning	1.58 (1.35–1.84)	1.63 (1.37–1.94)
Suicide behavior	1.53 (1.34–1.74)	1.66 (1.47–1.86)
Transport injuries	1.07 (1.00–1.14)	1.24 (1.16–1.32)
Traumatic brain injury	1.17 (1.10–1.26)	1.21 (1.12–1.31)
**Somatic diseases**		
Asthma	1.25 (1.20–1.30)	1.24 (1.18–1.30)
Autoimmune diseases	1.11 (1.00–1.24)	1.14 (1.03–1.26)
Bacterial infection	1.13 (1.09–1.17)	1.09 (1.05–1.13)
Epilepsy	1.34 (1.14–1.57)	1.50 (1.27–1.77)
Gastrointestinal autoimmune diseases	1.23 (1.04–1.45)	1.01 (0.87–1.18)
Obesity	1.44 (1.30–1.59)	1.31 (1.18–1.45)
Sleep disorder	2.29 (2.10–2.51)	2.37 (2.15–2.62)
Type 1 diabetes	0.87 (0.69–1.11)	1.16 (0.93–1.45)
Viral infection	1.15 (1.12–1.19)	1.12 (1.08–1.16)

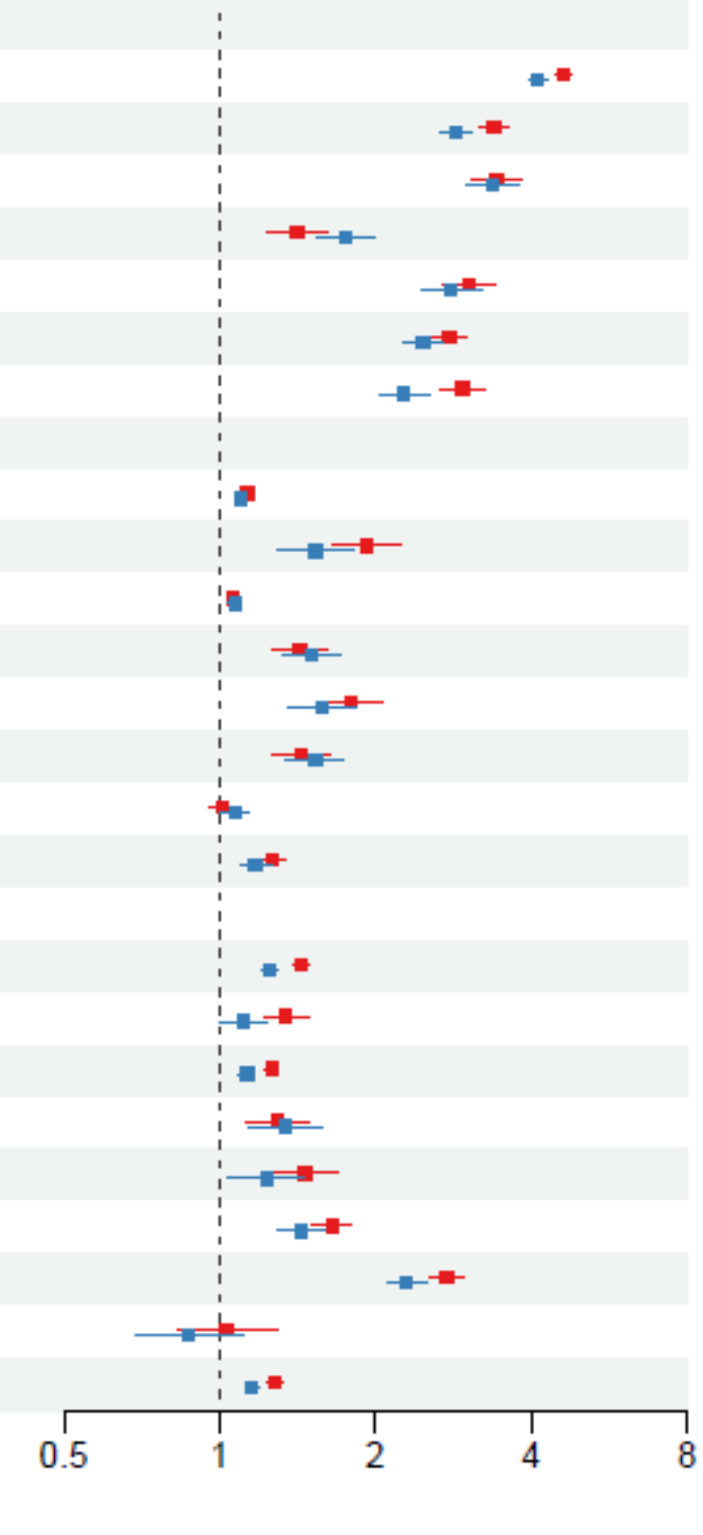

Supplementary Figure 3. Subgroup analysis: Associations between paternal ADHD and adverse offspring outcomes, by offspring sex.

Abbreviations: ADHD, Attention-Deficit/Hyperactivity Disorder.

Notation: Accidental injuries, falls, poisoning, suicide behavior, and transport injuries include both diagnoses and deaths; all other outcomes are based on diagnoses only. Models are adjusted for maternal and paternal education levels, as well as maternal and paternal psychiatric comorbidities. The reference group consists of offspring whose parents both do not have ADHD. After matching, 19,836 male offspring with any parental ADHD were matched to 286,168 male offspring without parental ADHD; 14,035 female offspring with paternal ADHD were matched to 134,279 female offspring without parental ADHD.

Outcomes	Both parents with ADHD – male	Both parents with ADHD – female
**Psychiatric disorders**		
ADHD	6.61 (5.96–7.33)	7.94 (6.89–9.14)
Autism	4.71 (4.02–5.53)	6.49 (5.07–8.30)
Conduct disorder	4.34 (3.30–5.70)	7.12 (4.83–10.49)
Intellectual disability	1.79 (1.30–2.44)	1.20 (0.76–1.91)
Tic disorder	3.57 (2.52–5.07)	4.08 (2.07–8.05)
Anxiety	3.84 (3.01–4.90)	3.07 (2.56–3.69)
Major depression disorder	3.15 (2.30–4.32)	2.59 (2.05–3.28)
**Behavioral outcomes**		
Accidental injuries	1.12 (1.05–1.20)	1.22 (1.14–1.32)
Assault/victimization	2.35 (1.62–3.41)	3.14 (2.02–4.87)
Falls	1.03 (0.95–1.12)	1.17 (1.07–1.28)
Intentional self–harm	1.86 (1.31–2.65)	1.99 (1.45–2.73)
Poisoning	2.46 (1.77–3.42)	3.03 (2.14–4.30)
Suicide behavior	1.89 (1.33–2.68)	1.99 (1.46–2.73)
Transport injuries	0.95 (0.80–1.13)	1.13 (0.93–1.37)
Traumatic brain injury	1.31 (1.11–1.55)	1.12 (0.91–1.37)
**Somatic diseases**		
Asthma	1.44 (1.29–1.60)	1.58 (1.38–1.81)
Autoimmune diseases	1.07 (0.78–1.46)	1.38 (1.04–1.83)
Bacterial infection	1.15 (1.04–1.26)	1.24 (1.12–1.36)
Epilepsy	1.22 (0.79–1.88)	1.85 (1.19–2.88)
Gastrointestinal autoimmune diseases	1.31 (0.81–2.11)	1.32 (0.87–1.99)
Obesity	1.20 (0.91–1.59)	1.51 (1.16–1.98)
Sleep disorder	3.41 (2.75–4.22)	3.40 (2.64–4.36)
Type 1 diabetes	0.93 (0.49–1.76)	2.10 (1.21–3.64)
Viral infection	1.22 (1.12–1.33)	1.32 (1.20–1.45)

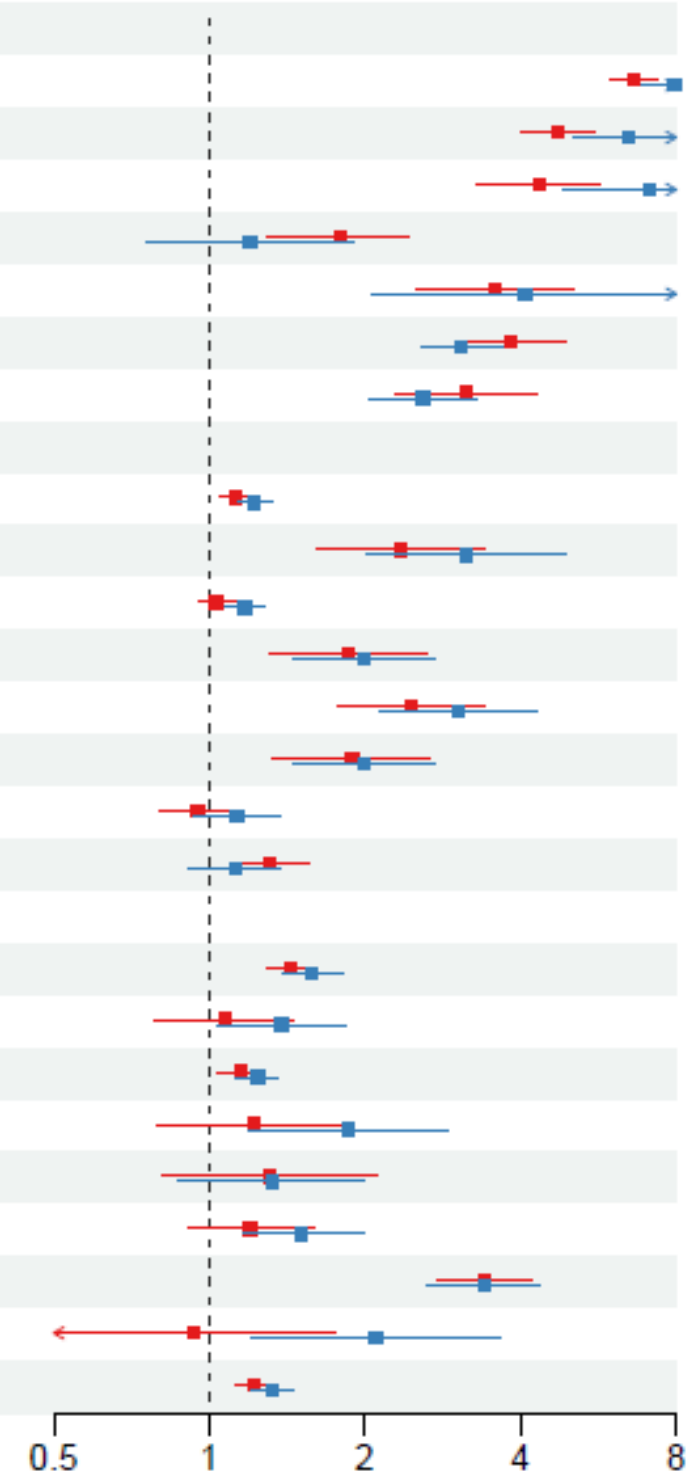

Sex of index person Male Female

Supplementary Figure 4. Subgroup analysis: Associations between parental ADHD (both parents) and adverse offspring outcomes, by offspring sex.

Abbreviations: ADHD, Attention-Deficit/Hyperactivity Disorder.

Notation: Accidental injuries, falls, poisoning, suicide behavior, and transport injuries include both diagnoses and deaths; all other outcomes are based on diagnoses only. Models are adjusted for maternal and paternal education levels, as well as maternal and paternal psychiatric comorbidities. The reference group consists of offspring whose parents both do not have ADHD. After matching, 2,278 male offspring with both parents having ADHD were matched to 21,345 male offspring without parental ADHD; 2,073 female offspring with both parents having ADHD were matched to 19,472 female offspring without parental ADHD.

Outcomes	Maternal birth year < 1980	Maternal birth year >= 1980
**Psychiatric disorders**		
ADHD	5.13 (4.97−5.28)	3.74 (3.60−3.89)
Autism	3.72 (3.54−3.91)	2.46 (2.30−2.63)
Conduct disorder	3.80 (3.45−4.18)	2.88 (2.58−3.22)
Intellectual disability	1.64 (1.47−1.82)	1.34 (1.18−1.51)
Tic disorder	3.02 (2.72−3.35)	2.56 (2.25−2.91)
Anxiety	2.66 (2.54−2.78)	2.17 (2.01−2.34)
Major depression disorder	2.48 (2.35−2.61)	1.78 (1.61−1.96)
**Behavioral outcomes**		
Accidental injuries	1.12 (1.10−1.14)	1.14 (1.12−1.16)
Assault/victimization	1.94 (1.74−2.16)	1.55 (1.34−1.80)
Falls	1.05 (1.02−1.07)	1.11 (1.09−1.14)
Intentional self−harm	1.54 (1.42−1.67)	1.41 (1.27−1.57)
Poisoning	1.67 (1.49−1.87)	1.54 (1.36−1.74)
Suicide behavior	1.54 (1.42−1.67)	1.42 (1.28−1.58)
Transport injuries	1.12 (1.07−1.16)	1.06 (1.00−1.12)
Traumatic brain injury	1.23 (1.17−1.29)	1.24 (1.17−1.31)
**Somatic diseases**		
Asthma	1.32 (1.28−1.37)	1.35 (1.31−1.39)
Autoimmune diseases	1.24 (1.16−1.33)	1.14 (1.05−1.24)
Bacterial infection	1.23 (1.20−1.26)	1.14 (1.11−1.18)
Epilepsy	1.39 (1.24−1.55)	1.30 (1.15−1.47)
Gastrointestinal autoimmune diseases	1.20 (1.09−1.33)	1.15 (1.01−1.30)
Obesity	1.73 (1.62−1.85)	1.30 (1.20−1.40)
Sleep disorder	2.62 (2.46−2.78)	2.26 (2.10−2.43)
Type 1 diabetes	0.98 (0.84−1.14)	1.10 (0.91−1.33)
Viral infection	1.28 (1.25−1.31)	1.17 (1.14−1.20)

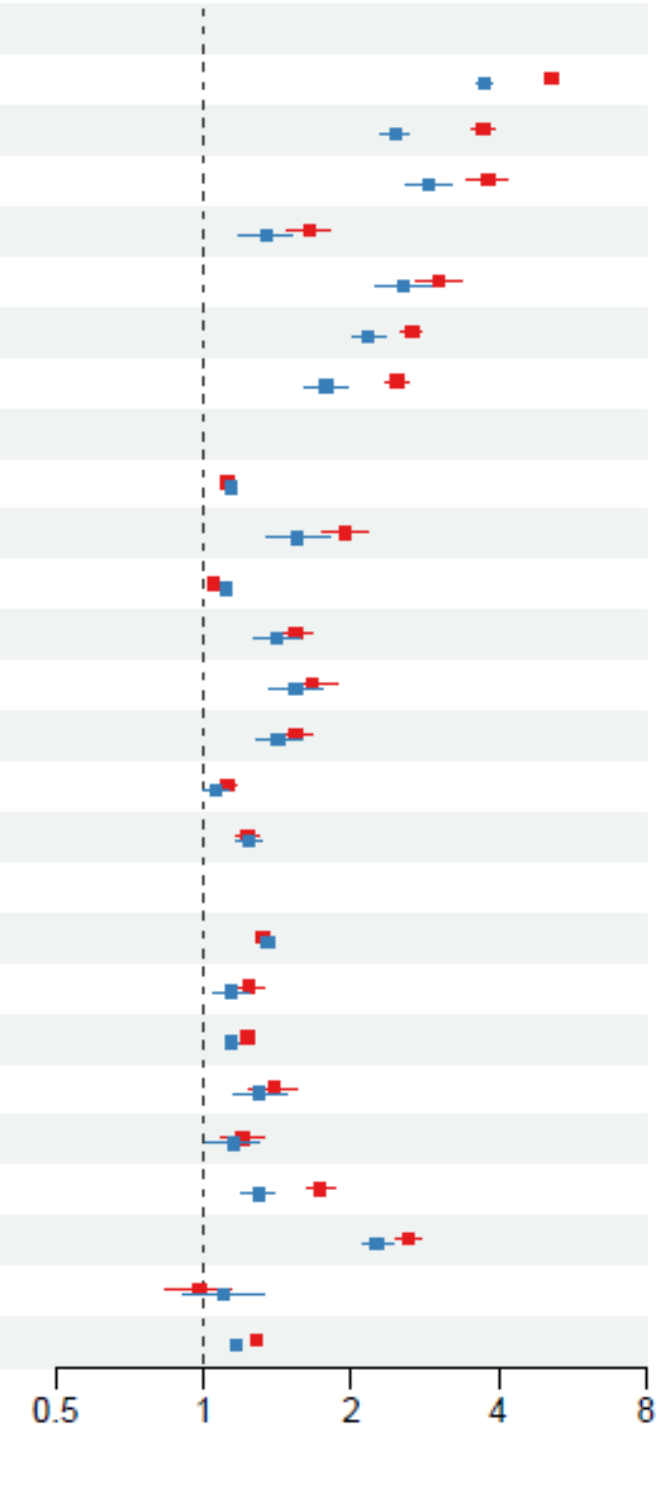

Supplementary Figure 5. Subgroup analysis: Associations between any parental ADHD and adverse offspring outcomes, stratified by maternal birth year (before 1980 vs. 1980 or later).

Abbreviations: ADHD, Attention-Deficit/Hyperactivity Disorder.

Notation: Accidental injuries, falls, poisoning, suicide behavior, and transport injuries include both diagnoses and deaths; all other outcomes are based on diagnoses only. Models are adjusted for maternal and paternal education levels, as well as maternal and paternal psychiatric comorbidities. The reference group consists of offspring whose parents both do not have ADHD. After matching, 29,753 offspring whose mother was born in 1980 or later with at least one parent with ADHD were matched to 282,332 offspring without parental ADHD; 27,099 offspring whose mother was born before 1980 with at least one parent with ADHD were matched to 262,384 offspring without parental ADHD.

Outcomes	Paternal birth year < 1980	Paternal birth year >= 1980
**Psychiatric disorders**		
ADHD	4.86 (4.73−5.00)	3.69 (3.51−3.87)
Autism	3.50 (3.34−3.67)	2.38 (2.19−2.58)
Conduct disorder	3.50 (3.21−3.82)	2.99 (2.62−3.41)
Intellectual disability	1.61 (1.47−1.77)	1.27 (1.09−1.47)
Tic disorder	2.97 (2.71−3.26)	2.47 (2.10−2.89)
Anxiety	2.63 (2.52−2.74)	2.04 (1.85−2.25)
Major depression disorder	2.35 (2.23−2.47)	1.89 (1.66−2.15)
**Behavioral outcomes**		
Accidental injuries	1.13 (1.11−1.14)	1.14 (1.11−1.16)
Assault/victimization	1.89 (1.71−2.08)	1.48 (1.22−1.80)
Falls	1.06 (1.04−1.08)	1.11 (1.08−1.14)
Intentional self−harm	1.56 (1.45−1.68)	1.32 (1.15−1.51)
Poisoning	1.70 (1.53−1.88)	1.46 (1.26−1.68)
Suicide behavior	1.56 (1.45−1.67)	1.33 (1.17−1.52)
Transport injuries	1.10 (1.06−1.15)	1.06 (1.00−1.14)
Traumatic brain injury	1.24 (1.19−1.30)	1.22 (1.14−1.30)
**Somatic diseases**		
Asthma	1.37 (1.33−1.41)	1.29 (1.24−1.34)
Autoimmune diseases	1.21 (1.14−1.29)	1.18 (1.06−1.30)
Bacterial infection	1.23 (1.20−1.26)	1.12 (1.08−1.16)
Epilepsy	1.38 (1.25−1.52)	1.29 (1.12−1.50)
Gastrointestinal autoimmune diseases	1.17 (1.06−1.28)	1.22 (1.05−1.42)
Obesity	1.66 (1.56−1.76)	1.25 (1.14−1.38)
Sleep disorder	2.57 (2.43−2.72)	2.21 (2.03−2.41)
Type 1 diabetes	1.00 (0.87−1.15)	1.12 (0.90−1.40)
Viral infection	1.27 (1.24−1.30)	1.15 (1.11−1.18)

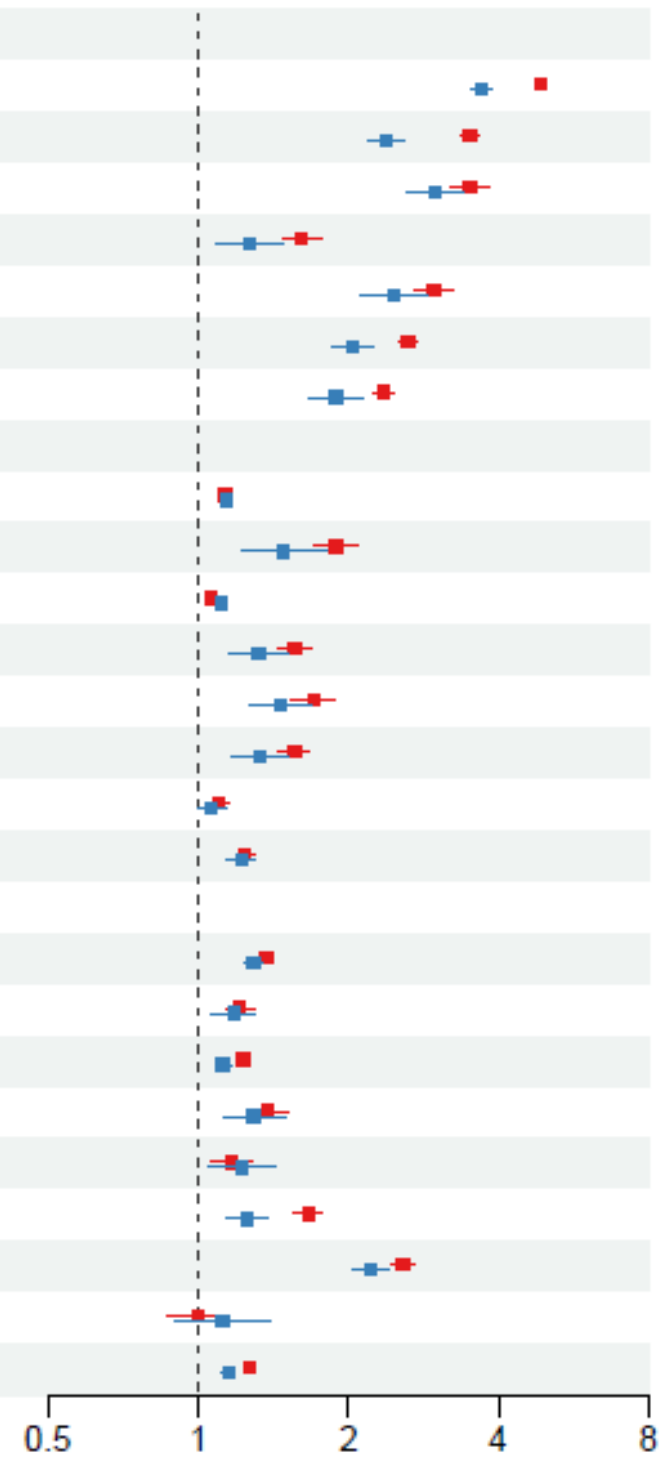

Supplementary Figure 6. Subgroup analysis: Associations between any parental ADHD and adverse offspring outcomes, stratified by paternal birth year (before 1980 vs. 1980 or later).

Abbreviations: ADHD, Attention-Deficit/Hyperactivity Disorder.

Notation: Accidental injuries, falls, poisoning, suicide behavior, and transport injuries include both diagnoses and deaths; all other outcomes are based on diagnoses only. Models are adjusted for maternal and paternal education levels, as well as maternal and paternal psychiatric comorbidities. The reference group consists of offspring whose parents both do not have ADHD. After matching, 22,595 offspring whose father was born in 1980 or later with at least one parent with ADHD were matched to 213,538 offspring without parental ADHD; 34,257 offspring whose father was born before 1980 with at least one parent with ADHD were matched to 331,178 offspring without parental ADHD.
